# Laser Emission of Thioflavin T Uncovers Protein Aggregation in Amyloid Nucleation Phase

**DOI:** 10.1021/acsphotonics.1c00082

**Published:** 2021-08-11

**Authors:** Piotr Hanczyc, Piotr Fita

**Affiliations:** Institute of Experimental Physics, Faculty of Physics, University of Warsaw, Pasteura 5, 02-093 Warsaw, Poland

**Keywords:** amyloid, amplified spontaneous emission, dye staining, tissue and CSF detection, neurodegeneration, lasing, stimulated emission

## Abstract

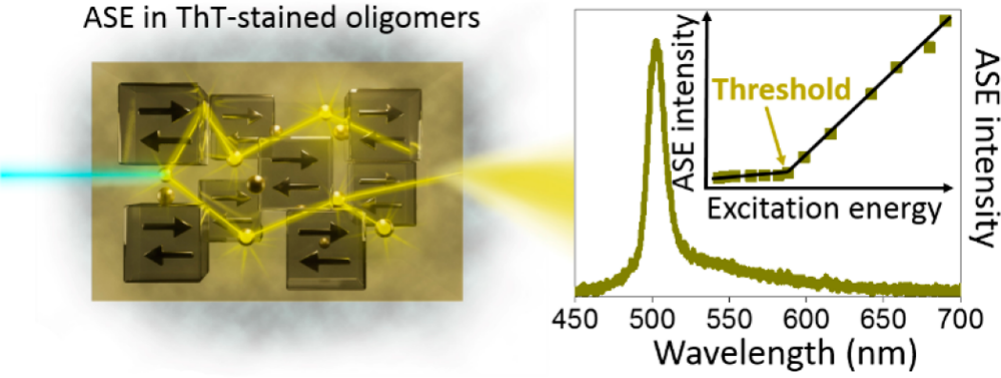

There is currently no definitive test for early detection of neurodegeneration which is linked with protein aggregation. Finding methods capable of detecting intermediate states of protein aggregates, named oligomers, is critical for the early stage diagnosis of over 30 neurodegenerative diseases including Alzheimer’s or Parkinson’s. Currently, fluorescence-based imaging using Thioflavin T (ThT) dye is the gold standard for detecting protein aggregation. It is used to detect aggregation in vitro and in various tissues, including the cerebrospinal fluid (CSF), whereby the disease-related protein recombinant is seeded with the patient’s fluid. The major drawback of ThT is its lack of sensitivity to oligomeric forms of protein aggregates. Here, we overcome this limitation by transferring a ThT–oligomer mixture into solid state thin films and detecting fluorescence of ThT amplified in the process of stimulated emission. By monitoring the amplified spontaneous emission (ASE) we achieved a remarkable recognition sensitivity to prefibrillar oligomeric forms of insulin and lysozyme aggregates in vitro, to Aβ42 oligomers in the human protein recombinants seeded with CSF and to Aβ42 oligomers doped into brain tissue. Seeding with Alzheimer patient’s CSF containing Aβ42 and Tau aggregates revealed that only Aβ42 oligomers allowed generating ASE. Thus, we demonstrated that, in contrast to the current state-of-the-art, ASE of ThT, a commonly used histological dye, can be used to detect and differentiate amyloid oligomers and evaluate the risk levels of neurodegenerative diseases to potential patients before the clinical symptoms occur.

Amyloid is a form of an erratic protein structure associated with numerous devastating diseases such as the Alzheimer’s, Parkinson’s, or Creutzfeldt–Jakob disease.^1−5^ One of the simplest methods of detecting amyloid protein fibrils relies on staining them with small organic molecules that become brightly fluorescent upon binding.^6−8^ Among amyloid-sensitive organic molecules, Thioflavin T (ThT) is the most common fluorophore, widely used by clinicians and research laboratories for validating amyloid fibrils formation.^9−13^ In the presence of fibrils, Thioflavin T exhibits hundreds-fold increase of the fluorescence quantum yield. This effect is explained by inhibition of the internal rotation of molecular segments (Figure 1a,b) in ThT molecules bound with fibrils, because this rotation is involved in the ultrafast non-radiative deactivation of unbound ThT.^14,15^ It is considered that the amount of the β-sheets dominating the fibrils structure is critical for the ThT fluorescence. No emission in the presence of native proteins or only scarce emission enhancement in the presence of prefibrillar oligomeric forms can be detected.^16−19^ The low sensitivity to oligomers was confirmed with numerous standard organic fluorophores that were used for staining amyloids.^20^ It makes fluorescence detection based on standard organic fluorophores and, in particular, ThT insensitive to the early protein aggregation stage which is considered to be the key phase, when toxic oligomers are associated with devastating developments in neurodegenerative diseases.^21^

**Figure 1:**
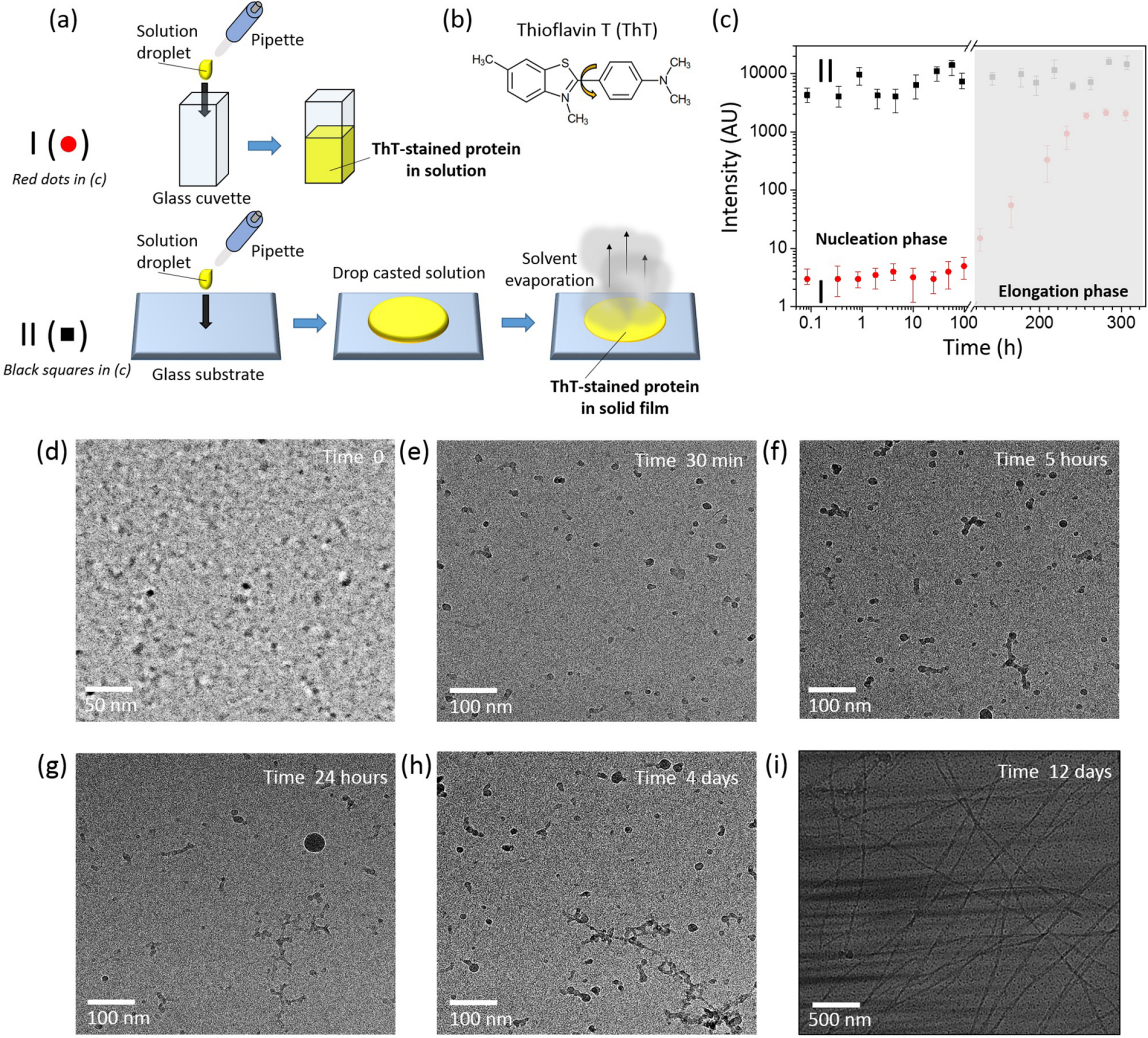
(a) Schematic illustration of sample preparation for fluorescence experiments: top panel represents preparation of solutions that correspond to red dots in the fluorescence intensity kinetics shown in graph (c), and bottom panel represents preparation of solid films that correspond to black squares in the fluorescence intensity kinetics graph (c). (b) Structure of Thioflavin T (ThT) with arrow indicating rotation of the molecular segments. (c) The fluorescence intensity kinetics of ThT in the presence of the aggregating lysozyme protein. Red dots show the fluorescence intensity recorded in solution and black squares represent data collected for the ThT–protein mixture films drop-casted on glass slides at specific time intervals. The samples for measurements were prepared with 0.21 mM ThT and 1.5 mM lysozyme protein solutions in a pH 2 buffer. (d–i) Transmission electron microscopy (TEM) images of the molecular species formed over time at pH 2 and 65 °C: (d) A uniform population of lysozyme protein monomers at time 0 (before heating); (e) sample after 30 min of incubation containing a large amount of small lysozyme oligomers; (f) larger oligomeric structures formed after 5 h; (g) image after 24 h and (h) after 4 days of incubation showing mixture of oligomers and protofibrils; (i) mature lysozyme fibrils formed after 12 days.

As a solution to that problem, many new dyes were designed to bind specifically to amyloid oligomers.^22^ The chemically modified ThT variants were also proposed for improving the detection of the intermediate aggregate species.^23,24^ Together with the chemical advancements, the fluorescent-based detection techniques for capturing different amyloid states were developed. ThT was used in the fluorescence correlation spectroscopy to detect amyloid plaques in blood.^12^ The time-resolved fluorescence was shown to be more sensitive toward early stage aggregates than standard steady-state fluorescence.^25^ The deeper understanding of the oligomers heterogeneity was achieved by single-molecule methods.^26,27^

Simultaneously to the chemical advancements and technical developments the ThT-based protocols were extensively refined. The gold standard imaging using ThT was implemented in studies on patient’s fluids whereby the problem of the low concentration of pathological proteins in biological fluids was solved by multiplication of aggregates in the misfolding cyclic amplification process or real-time quaking-induced conversion.^28,29^

In this Article, the fluorescence of ThT was amplified in the process of the stimulated emission. It was achieved by the change of the optical excitation from a simple lamp to a pulsed laser. We have demonstrated that measurements of the emitted light intensity for ThT-stained samples could be used for a more accurate detection of oligomeric amyloids from the very beginning of the protein aggregation. The simple extension can be applied into existing methods and protocols to study toxic species and get a deeper insight into protein aggregation pathways at the prefibrillar stage.

## Results

### Identification of Aggregated Species by Electron Microscopy and Infrared Spectroscopy

To identify the molecular species formed over time, attenuated total reflectance Fourier-transform infrared spectroscopy (ATR-FTIR)^30^ and transmission electron microscopy (TEM)^31^ were used (Figures 1 and S1). To probe the aggregated species, the native lysozyme was dissolved in a pH 2 buffer and incubated at an elevated temperature of 65 °C that is a standard denaturing environment for protein aggregation in vitro in test tubes.^32^

The ATR-FTIR measurements that were carried out in drying droplets showed that solvent evaporation has little or no influence on the protein secondary structure (Figure S1b). The infrared reflectance spectra revealed that lysozyme was already aggregated in films deposited from the solution incubated for 15 min, which was manifested by ∼25 cm^–1^ spectral shift in the amide I band, from 1648 cm^–1^ (corresponding to α-helices) to 1622 cm^–1^ (corresponding to β-sheets). The β-sheet content gradually increased in samples heated for longer periods of time but a clear differentiation between oligomers and fibrils was not possible using the FTIR spectroscopy. To visualize molecular species, TEM images were taken at specific times of protein incubation. Figure 1d shows the image of the freshly dissolved protein (time 0). Globular structures seen in panels e–h of Figure 1 indicate that oligomers are formed between 15 min and 4 days of incubation. After 24 h, first elongated structures started to appear, indicating the initiation of protofibrils formation. Long incubation in denaturing conditions lead to the formation of mature fibrils that are shown in Figure 1i.

### ThT Fluorescence in Solutions and Solid Thin Films

Fluorescence spectra in solutions and solid films were studied for ThT mixed with protein monomers, oligomers and fibrils prepared of lysozyme. The standard fluorescence intensity kinetics recorded for ThT in solution mixed with the lysozyme protein is shown in Figure 1c. It corresponds very well to the ATR-FTIR spectra and TEM images that were used to determine the molecular species of lysozyme aggregates. The fluorescence is very weak during the first 4 days of incubation in the pH 2 buffer at an elevated temperature of 65 °C. The visible rise of the emission intensity can be detected only after approximately 100 h of incubation. The ThT kinetics in solution show that the first 4 days can be associated with the so-called nucleation phase when lysozyme monomers form early stage aggregates–the oligomers. After approximately 100 h, the lysozyme oligomers enter the elongation phase and mature fibrils are formed after 12 days of continuous incubation at 65 °C (Figure 1i).

An experiment parallel to the measurement of the fluorescence kinetics in solution was performed with films that were drop-casted on glass slides at specific time intervals corresponding to different stages of the protein aggregation (black squares in Figure 1c). Stationary fluorescence studies revealed that in the solid state proteins prevent crystallization of the dye, which leads to a significant enhancement of the ThT fluorescence.

In thin films stained with ThT, it was possible to record the fluorescence spectrum of the dye already at the prefibrillar nucleation phase when the fluorescence intensity of a liquid sample was still negligible. However, the ThT fluorescence in thin film samples had a similar intensity at each aggregation stage, thus, limiting the potential for recognition of specific aggregation forms by traditional fluorescence spectroscopy.

### Amplified Stimulated Emission (ASE) of ThT

**Figure 2:**
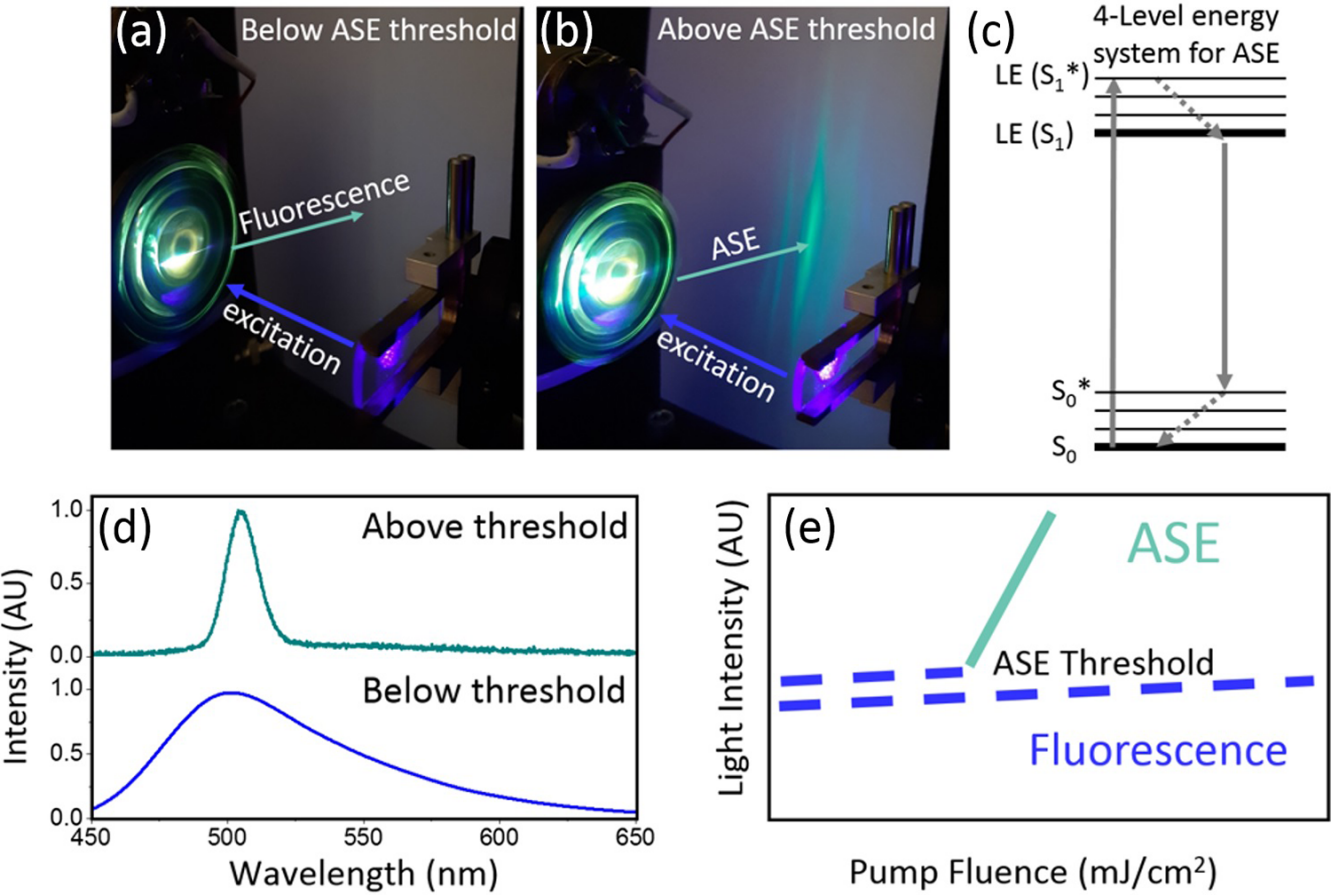
(a, b) Visual manifestation of fluorescence and ASE, respectively. A paper screen has been put in place of the spectrometer used to record ASE spectra. The excitation beam is generated by a femtosecond laser system and focused with a cylindrical lens into a narrow strip of light at the sample position. (c) Jablonski diagram of the four-level energy system in ThT, which enables ASE from the locally excited (LE) state, and (d) ThT ASE (top curve) and ThT fluorescence (bottom curve) spectra in the presence of aggregated lysozyme. (e) Schematic illustration demonstrating that the intensity of ASE exhibits a threshold behavior with respect to the excitation intensity in contrast to fluorescence.

We have discovered that this lack of structural sensitivity of fluorescence-based methods using ThT can be bypassed by studying the amplified spontaneous emission (ASE) of ThT bound to studied proteins instead of just recording its fluorescence spectrum. In the ASE process photons emitted spontaneously by excited molecules are multiplied in the stimulated emission process when they interact with other excited molecules during their propagation through the medium (it is the physical mechanism that underlies the operation of lasers). The result is a directional emission of high intensity light with its spectrum significantly narrower than that of fluorescence (Figure 2a–d).

In order to verify whether ASE can be generated with ThT embedded in a solid medium and optimize experimental parameters we initially used poly(vinyl alcohol) (PVA) as a well-defined environment, which is simpler than that consisting of solidified proteins. We have selected PVA for these experiments because it is a water-soluble polymer that can be used to prepare films of a sufficient optical quality. PVA can immobilize ThT molecules but, unlike proteins, does not participate in specific interactions with ThT.

During these preliminary experiments the optimal dye concentration in solution for investigation of the ASE generation was determined to be approximately 0.2 mM. Therefore, the ThT concentration of 0.21 mM was used for the following ASE experiments if not otherwise stated. For details of the preliminary experiments with ThT in PVA and optimization of sample parameters for the protein investigation see the Supporting Information and Figures S2–S5 therein.

The key discovery presented in this article is the ability of optical detection of prefibrillar oligomeric forms of proteins by studying ASE of samples stained with ThT. A prerequisite for ASE is the population inversion in light-amplifying molecules (i.e., more molecules must occupy the excited state than the ground state (Figure 2c). Because of the very efficient non-radiative deactivation of unbound ThT molecules the population inversion can be obtained only in ThT interacting with the host protein. Thus, ASE is intrinsically insensitive to unbound ThT molecules, whose excited-state lifetime is too short to allow population inversion.

In contrast to fluorescence, ASE appears only when intensity of the exciting light exceeds a certain threshold which is dictated by the competition between the energy gain due to stimulated emission and energy losses, for instance, due to light scattering.^33,34^ The latter is linked to the microscopic structure of the medium. In particular, the progressing protein aggregation increases the yield of light scattering.^35^ This fact makes the ASE spectrum and threshold the parameters, which are directly related to the aggregated form of the protein. Therefore, ASE provides additional bioanalytical information that complements traditional fluorescence methods.^36−39^

So far, no studies of ASE with ThT have been carried out to identify and detect amyloids and, in particular, amyloid oligomers. As we show, ASE-based analysis is sensitive to protein organization and it reveals ThT interactions with oligomers already in the nucleation phase of protein aggregation. In this work, ASE of ThT-stained proteins has been detected in solid thin films drop-casted from solutions containing amyloids prepared in test tubes, brain tissue doped with amyloid oligomer phantoms, and from human protein recombinants seeded with CSF. In our experimental setup, thin film samples were typically excited with a beam of short laser pulses focused with a cylindrical lens into a narrow stripe (see the experimental setup in the Supporting Information, Scheme 1). In order to determine the ASE threshold the excitation (pump) energy was gradually increased and at the same time the intensity and spectrum of the emitted light were recorded with a spectrometer. Crossing of the ASE threshold was indicated by narrowing of the emission spectrum (Figure 2d) and the increasing slope of the dependence of the emission intensity on the excitation intensity (Figure 2e); in this work the excitation intensity is expressed in terms of the average pump fluence, that is, the energy of the pump pulse per unit area of the laser beam in focus. For several samples studied in this work, the maximum excitation intensity obtainable with a cylindrical lens was too low to reach the ASE threshold. In such cases, the cylindrical lens was replaced with a spherical one, which resulted in a smaller area of the focal point and a higher pump fluence.

Four proteins were examined in order to verify the applicability of ASE to study protein aggregation: insulin, lysozyme, Aβ42, and Tau. Insulin was used because, in aqueous solutions, it forms amyloid fibrils at low pH, but it retains the α-helical structure at high pH.^40^ Thus, it can be conveniently used to demonstrate the sensitivity of ASE to amyloid formation. Lysozyme was chosen for a more detailed analysis because of its very slow aggregation kinetics that usually lasts a few days before mature fibrils are formed.^41^ Human protein recombinants, Tau and Aβ42 were seeded with Alzheimer patient’s CSF samples and examined in the context of the detection of early stage aggregates in patient’s body fluids.

### Discrimination between Protein Monomers and Aggregates Using ASE

ASE studies of insulin were performed in thin films drop-casted from aqueous solutions prepared at pH 2 and 12. The formation of insulin fibrils at acidic pH was confirmed by ATR-FTIR spectroscopy. The main peak in the amide I region is located at 1631 cm^–1^, which is associated with the β-sheet structure in insulin aggregates (Figure S6). In contrast, at pH 12, insulin does not aggregate due to the electrostatic repulsion, and it retains the α-helical structure.^40^ The band of insulin monomers in the infrared spectrum has its maximum at 1653 cm^–1^, which corresponds to the α-helix^42^ (Figure S6).

ASE in the insulin thin films was detected in samples containing protein aggregates drop-casted from solutions prepared at pH 2, whereas negligible fluorescence and no spectral evidence of ASE was observed for samples with protein monomers drop-casted from pH 12 solutions (Figure S7). This result confirms that ASE, alike stationary fluorescence, has a high sensitivity for the β-sheet motif, which is richly represented in the aggregated form of insulin and other amyloid forming proteins.

### ASE Recognition of Lysozyme and Aβ42 Oligomers

**Figure 3:**
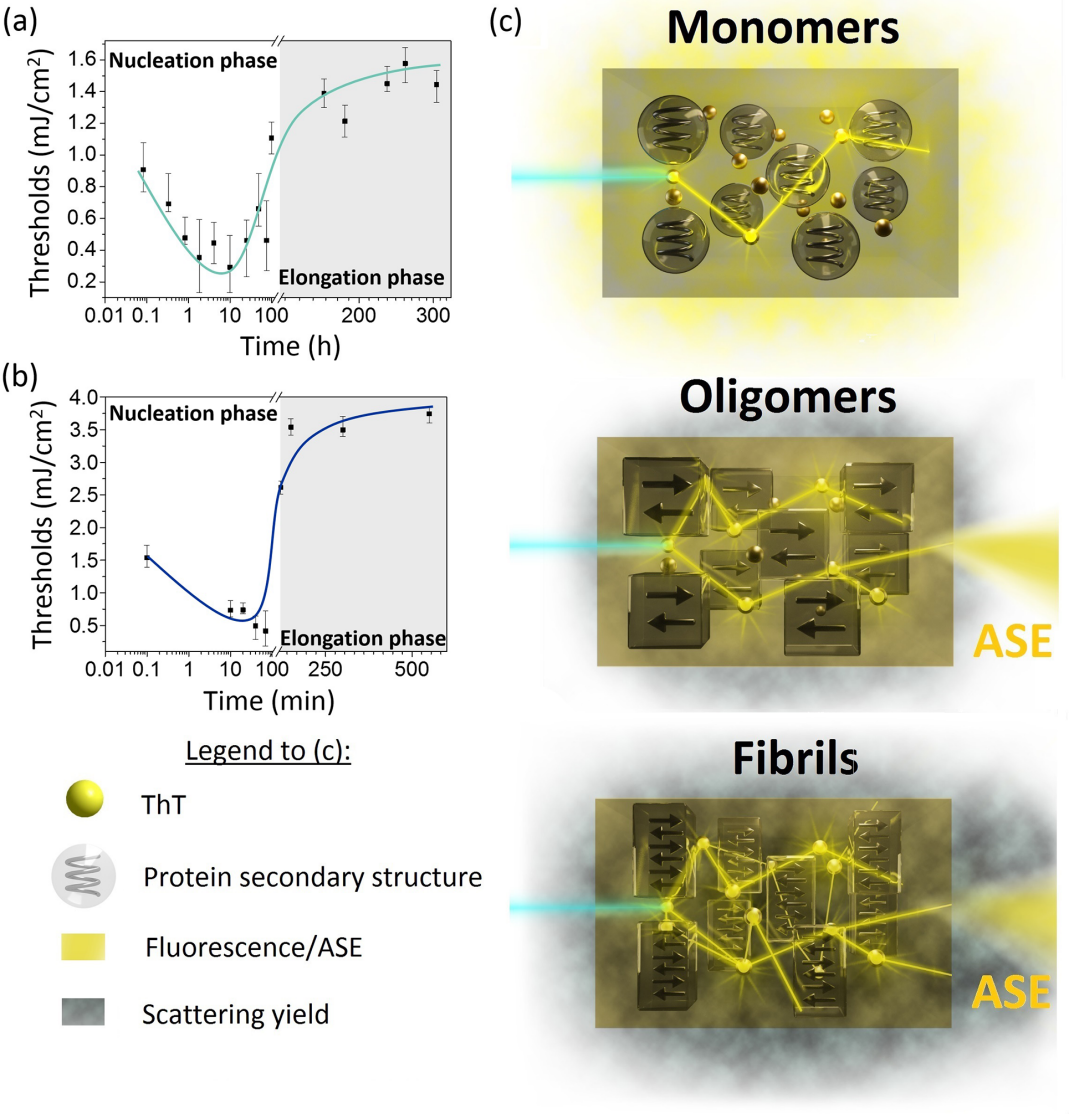
Kinetics of the ASE threshold for ThT-stained (a) lysozyme and (b) Aβ42 samples monitored at specific times. The graph is divided into two sections: left, the nucleation phase when prefibrillary aggregates are formed; and right, the elongation phase when protofibrils and mature fibrils grow. Black squares represent the ASE thresholds for the ThT concentration of 0.2 mM calculated by averaging results of three independent experiments and the cyan line indicates the trend over the time frame from 10 min to 13 days for lysozyme and from 10 to 550 min for Aβ42. (c) Schematic drawing of ASE generation in the presence of different protein forms: monomers (top, only extremely weak fluorescence can be detected (marked as the yellow cloud), oligomers (middle, ASE at a low threshold), and fibrils (bottom, ASE at a high threshold). The gray scale represents the yield of scattering, which is increasing with the progressing aggregation.

Figure 3a shows ASE thresholds for the ThT/lysozyme mixture drop-casted at specific times of incubation at 65 °C, that correspond to different protein aggregation phases. The data points were collected at the same time frame as the solution and solid film kinetics presented in Figure 1c. The starting point in Figure 3a corresponds to the ASE threshold measured for ThT with the lysozyme protein drop-casted at ambient conditions, which was equal to 0.9 mJ/cm^2^. The second point corresponds to the ASE threshold measured in the lysozyme protein incubated at 65 °C for 15 min, which turned out to be lower and equal to 0.75 mJ/cm^2^. Also, the following measurements showed a decreasing trend of ASE thresholds within the first hour of the protein aggregation. After the first hour the threshold level stabilized and remained in the range of 0.3–0.7 mJ/cm^2^ until the third day of incubation. The initial 72 h are within the nucleation phase of the protein aggregation.

Drop-casting the lysozyme heated for 4 days at 65 °C revealed a rise of the ASE threshold up to 1.1 mJ/cm^2^ and following measurements showed a further increase of the threshold. The standard fluorescence kinetics recorded in solution (Figure 1c) indicates that day four (>100 h) is the transition stage between nucleation and elongation phases of the protein aggregation, when oligomers start to form larger structures named protofibrils. The elongation phase lasts until the self-interwinding protofibrils form mature fibrils. According to the ThT kinetics test in solution, electron microscopy, and infrared spectroscopy, mature fibrils were formed after 10 days of incubation at 65 °C (Figures 1 and S1). Fibrils are the final product of the protein aggregation and they are considered to be a structurally stable form. The ASE threshold for mature fibrils drop-casted on the 10th day was measured to be 1.5 mJ/cm^2^ and it was constant on the following days with a small deviation of ±0.1 mJ/cm^2^. The experiment was repeated three times. ASE thresholds measured during each experimental run followed the same trend, with deviations of thresholds measured at the same incubation times not exceeding 0.2 mJ/cm^2^ (Figure S8(a)).

The dependence of ASE spectra on the excitation intensity in ThT-stained lysozyme oligomers is presented in Figure S9. The full width at half-maximum (fwhm) of the ASE band decreases with the increasing pump fluence, and it also depends on the lysozyme oligomers structure. The fwhm was between 11 and 13 nm at the pump fluence of 2.6–3.8 mJ/cm^2^ for the early stage oligomers drop-casted from the solution incubated at 65 °C for 15 min. The lysozyme drop-casted after 10 h of incubation in the same conditions had the ASE band broader than 14 nm fwhm at the pump fluence of 2.8 mJ/cm^2^ or higher. The data indicates that the growth of aggregates over time results in the broadening of ASE spectra. However, the interpretation of the correlation between the ASE spectral width and the structure of protein aggregates is not trivial and will require modeling in order to take into account also the scattering effects.^43,44^ Thus, in the next section of this article the relationship between the structure of the aggregates and ASE will be discussed only in terms of ASE thresholds.

Measurements analogous to the experiments with lysozyme were carried out with the Aβ42 peptide, which exhibits a much faster aggregation dynamics. Fluorescence kinetics recorded in solution revealed that Aβ42 aggregated within 2 h of incubation at 37 °C (Figure S10). The nucleation phase lasted only 90–100 min and was followed by the short elongation phase. The latter lasted up to 110–150 min from the start of the incubation. The fluorescence intensity reached a plateau after 180 min from the start, which indicated the formation of mature fibrils.

Based on the aggregation dynamics of Aβ42 in solution, thin films drop-casted at 10, 20, 40, and 70 min (counting from the start of incubation at 37 °C) corresponded to the nucleation phase of Aβ42 (examples of the ASE and FTIR spectra of ThT-stained Aβ42 oligomers in thin films are presented in Figure S11). Thin films made between 120 and 150 min after the start of the incubation corresponded to the elongation phase and those drop-casted after 180 min contained mature fibrils (Figure S10). ASE thresholds for the Aβ42/ThT mixture drop-casted at different times showed the same trend as in the case of lysozyme. There was a characteristic decrease of the ASE thresholds in the nucleation phase (10–70 min), which was followed by a significant increase of the ASE thresholds when in the elongation phase (>180 min) (Figure 3b). The experiment was repeated three times (Figure S8(b)), showing good reproducibility of the trend.

The above-described experiments with lysozyme and Aβ42 demonstrate a remarkable advantage of ASE over fluorescence: values of the ASE threshold measured during the nucleation phase (before oligomers merge into fibrils) decrease over time in a way that reflects the progressing degree of aggregation. In contrast, ThT fluorescence cannot be even detected during this phase. Therefore, not only ASE can help to discriminate between monomers and oligomers, but kinetics of the ASE threshold can be used to follow the progress of oligomerization.

### Underlying Mechanism: Competition between Amplification and Scattering of Light

The observed difference of the ASE thresholds and spectra seen for protein aggregates (Table 1) in solid films can be explained in terms of the competition between energy gain and losses in the ASE generation process. The gain depends on the population of ThT molecules in the excited state, which in the first place is controlled by the excitation intensity. Nevertheless, aggregation changes the number of protein-bound ThT molecules in which the ultrafast non-radiative relaxation is hindered and have the excited-state lifetime long in comparison to that of the free dye molecules (as can be seen, for instance, in Figure S4(b) in the SI). As a consequence, the progressing aggregation should increase the gain.

**Table 1:** ASE Thresholds for Lysozyme and Aβ42 Aggregates

	ASE threshold (mJ/cm^2^)	
protein	oligomers	fibrils
lysozyme	0.3–0.9	1.1–1.5
Aβ42	0.5–1.5	2.7–3.5

On the other hand, the size of oligomers and fibrils formed during the incubation of the protein solutions is much larger than the size of protein monomers. The aggregates are therefore much stronger light scatterers than monomers and it has been already well-known that the progressing protein aggregation affects the yield of light scattering.^35^ We have verified that the light scattering also plays a role in the studied protein films. To this end, we have built a simple experimental setup for the observation of the coherent backscattering (CBS) of light^45,46^ (Figure S12(a)) and used it to test films made of lysozyme at selected stages of the aggregation. The CBS spot could be well seen on the incoherent scattering background for lysozyme oligomers and fibrils (Figure S12(b)), whereas it was indistinct for the protein monomers. As expected, the angular dependence of the scattered light intensity clearly shows the difference in the light scattering properties between protein monomers and aggregates (Figure S12(c)). Comparison of the CBS curves for various aggregates (Figure S12(d)) shows that the curves become narrower with the progressing aggregation. It means that the mean free path of a photon in the film becomes longer as the aggregates grow.^46^ After fibrils are formed, no further significant changes can be seen. The effect can be easily understood in terms of the amyloid formation: large fibrils are formed when smaller oligomers stick together and when the aggregates grow, their number decreases. Larger aggregates have a larger cross section for light scattering, but at the same time the mean distance between the scatterers increases, effectively leading to a longer mean free path of a photon.

Altogether, the aggregation affects the generation of ASE through a number of parameters: the effective gain, the cross section for light scattering by aggregates, and the mean free path of a photon in the film. In order to quantitatively reproduce the dependence of ASE thresholds on the form of protein aggregates a complex model taking into account all the above-mentioned effects would be required.^43,44,47^ At this stage, however, it can be concluded that the observed behavior of the ASE thresholds and spectra is the resultant of light amplification by excited ThT molecules and light scattering by protein aggregates, as schematically illustrated in Figure 3c.

The fact that ASE reflects the competition between the light amplification and scattering makes it a powerful bioanalytical tool for studying protein aggregation, which complements the state-of-the-art methods, the ThT fluorescence assay and the Dynamic Light Scattering. These standard biophysical methods are insensitive to the nucleation phase of proteins due to the weak fluorescence of ThT or scarce scattering of oligomers, respectively. In contrast, monitoring ASE thresholds in thin films of ThT-stained proteins shows that optical detection of the intermediate oligomer species is possible, but only when light amplification is combined with scattering, such as in the ASE process.

### ASE in Biological Samples

In order to verify whether the ASE-based detection is a valuable analytical technique that can be applied to uncover protein oligomers in real biological samples, bovine brain tissue and CSF collected from patients with the Alzheimer’s disease were investigated. The brain tissue of a healthy animal was selected for these experiments in order to verify whether ASE can be generated in a strongly scattering medium of biological origin and will preserve its sensitivity to oligomers when the latter are prepared externally and mixed with the tissue. On the other hand, experiments with CSF were intended to answer the question if ASE can be the basis of future diagnostic tests for detection of amyloid oligomers in patients’ CSF. Two types of biosamples were prepared. First, thin films were made of pristine tissue and dried pristine CSF stained with ThT. Second, ThT-stained Aβ42 oligomers prepared earlier in test tubes were mixed with either brain tissue or CSF in order to obtain phantom samples with a high content of amyloids.

**Figure 4:**
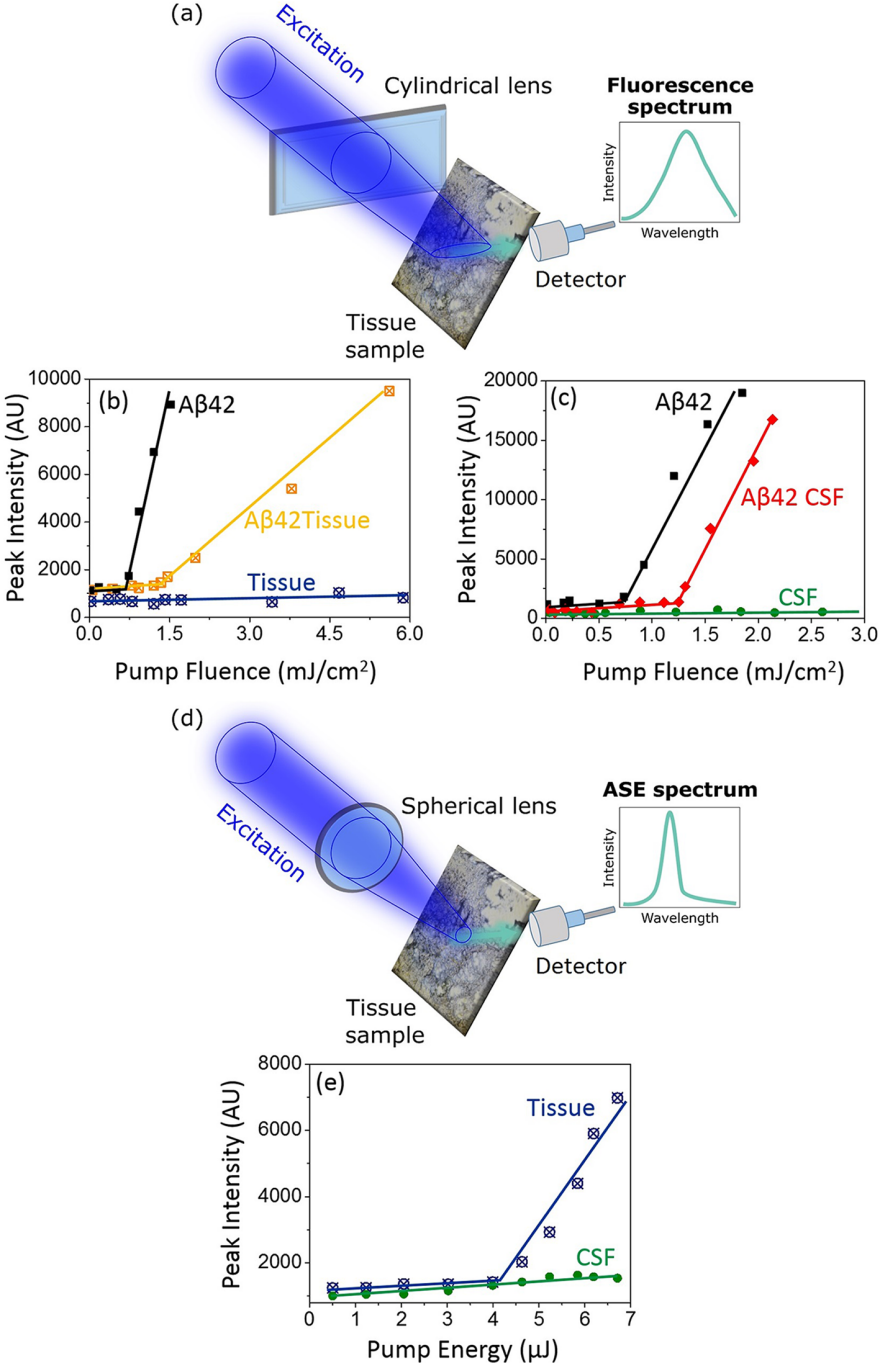
(a) Schematic drawing of biosamples excited through a cylindrical lens. (b) Dependence of the intensity of the emitted light on the excitation energy (pump fluence) used for the determination of the ASE thresholds in ThT-stained Aβ42 oligomers (black) and homogenized tissue with Aβ42 amyloid oligomer phantoms (orange) recorded in the setup configuration with the cylindrical lens; for tissue doped only with ThT (blue), no ASE was detected. (c) Dependence of the intensity of the emitted light on the excitation energy in the setup configuration with the cylindrical lens in ThT-stained Aβ42 oligomers (black) and CSF mixed with Aβ42 (red); no ASE was detected in CSF doped only with ThT (green). (d) Schematic drawing of biosamples excited through a spherical lens. (e) Dependence of the intensity of the emitted light on the excitation energy in the setup configuration with the spherical lens used for the determination of the ASE thresholds for ThT-stained homogenized pristine tissue. The excitation beam is generated by a femtosecond laser system. No ASE was recorded in ThT-stained pristine CSF.

In all experiments described above, the excitation beam was focused with a cylindrical lens that formed a narrow stripe of the excitation light on the sample (Figure 4a). In this configuration ThT-stained thin films of pristine tissue and pristine CSF exhibited only fluorescence, and ASE could not be observed. Therefore, phantom samples containing artificially prepared Aβ42 oligomers were tested. For both types of phantom samples containing Aβ42, ASE was generated and the dependence of the emitted light intensity on the excitation energy showed a characteristic threshold (Figure 4b,c). The ASE thresholds measured for Aβ42 oligomers mixed with both biomaterials were higher than for pristine Aβ42 oligomers due to the additional scattering introduced by the tissue and CSF in the dry state.

Next, the experiments were repeated, whereby the cylindrical lens was replaced with a spherical one in order to reduce the size of the focal point and obtain a higher pump fluence (Figure 4d). The higher excitation intensity resulted in the generation of ASE in the ThT-stained pristine tissue (Figure 4e). This fact indicates that tissue prevents the dye crystallization in the solid state, and if the excitation energy is high enough, it is possible to generate ASE in bovine brain tissue even without amyloid phantoms. This is inconvenient in terms of analyzing protein aggregation by ASE because, apparently, the signal may arise from ThT interacting with either amyloids or tissue.

In the case of CSF doped with ThT and drop-casted on a glass slide, no ASE was recorded, even in the experimental configuration with the spherical lens. The reason is the low concentration of proteins in CSF, of the order of ng/L (Table S4), whereas the ThT concentration required for the ASE generation is orders of magnitude larger, in the range of mg/L. Thus, the dye excess crystallizes and ASE cannot be induced in pristine CSF.

The experiments described in this section proved that ASE can be induced in biological amyloid-containing samples and that the ASE-based analysis can be particularly useful for the detection of amyloids in CSF if only their concentration is sufficient.

### ASE in Proteins Seeded by CSF

The problem of the low concentration of pathological proteins in CSF can be solved by seeding the appropriate human protein recombinant with body fluid, which multiplies disease-related aggregates in the protein misfolding cyclic amplification process.^48^ This approach was previously used to multiply pathogenic prion aggregates, whereby the recombinant PrP protein was mixed with the CSF of a patient having the Creutzfeldt–Jakob disease (CFD),^11^ α-synuclein was combined with CSF collected from patients with the Parkison’s disease,^29^ or the Tau protein was added to CSF of patients with the Pick disease.^49^ It is therefore expected that seeding Aβ42 monomers with CSF containing Aβ42 oligomers will also result in the multiplication of aggregates through the protein misfolding cyclic amplification; however, this approach has not been applied yet.

At this point it is worth noting that, for the development of Alzheimer’s disease, both the Tau protein forming neurofibrillary tangles^50^ and amyloid-β (Aβ) forming senile plaques are considered to be the key proteins involved in the initiation of neurodegeneration. Analysis of Aβ aggregation is particularly challenging because Tau and Aβ proteins are known to interact and they influence each other’s aggregation pathways.^51,52^ Thus, in order to verify if ASE-based methods may help to reveal the role of Tau and Aβ in the neurodegeneration, we carried out experiments with both proteins seeded with CSF of an Alzheimer patient. Mixtures of the appropriate protein (Tau or Aβ) with ThT and CSF were incubated at 37 °C, and thin films were drop-casted at specific times of incubation. Tau seeds present in the CSF should bind the Tau recombinant protein, whereas analogues of amyloid-β present in the CSF should bind Aβ42. As a result, pathogenic aggregates should be multiplied.

The results turned out to be very promising. ASE was not recorded for samples with the Tau protein seeded with CSF or 2 weeks old aggregated Tau (with no addition of CSF). In contrast, samples containing Aβ42 seeded with CSF exhibited ASE. Moreover, experiments carried out with various CSF samples revealed that ASE thresholds were strongly dependent on the CSF used for the seeding. Two important conclusions can be drawn from these studies of CSF-seeded proteins. First, ASE can be used to differentiate between Tau aggregates and amyloid-β oligomers. Second, amyloids can be detected in CSF by the ASE-based methodology when the protein misfolding cyclic amplification is applied to the sample. Below, we further investigate the potential of this concept.

### ASE in ThT-Doped Aβ42-Seeded with CSF of Alzheimer Patients

**Figure 5:**
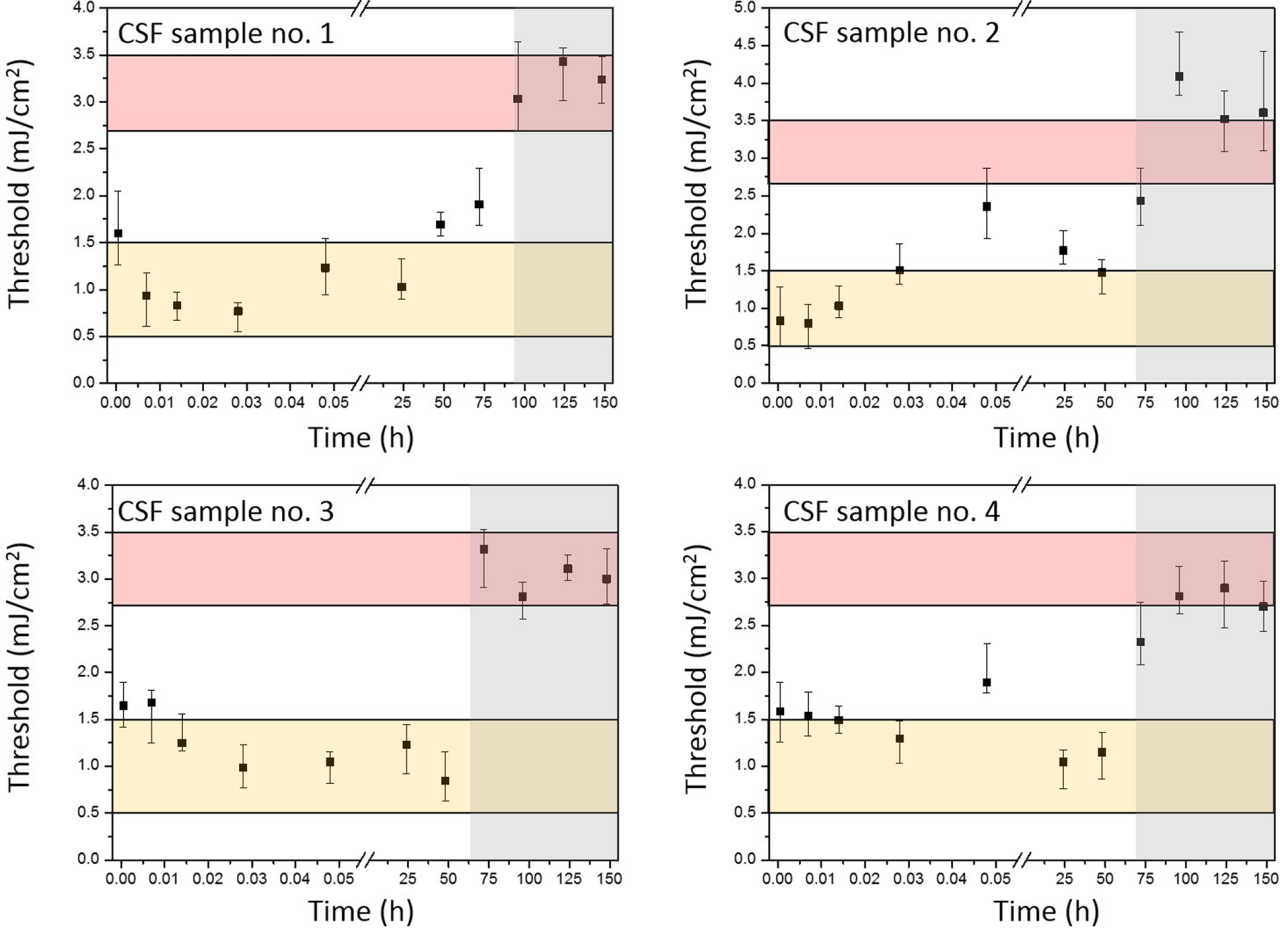
Kinetics of the ASE thresholds for ThT-stained Aβ42 seeded with four different CSF samples collected from patients with diagnosed Alzheimer disease. Each graph is divided into two sections: left, with white background, the nucleation phase when prefibrillary aggregates are formed, and right, with gray background, the elongation phase when fibrils grow. The ranges of ASE thresholds for thin films made of pristine Aβ42, which is the reference for seeded Aβ42, are marked with colored boxes: yellow, corresponding to the ASE thresholds measured in the nucleation phase for oligomers, and red, corresponding to the ASE thresholds in the elongation phase for fibrils. Black squares represent average ASE thresholds measured at a given time in three independent experiments. The error bars represent the range of values measured in these experiments.

Figure 5 shows the temporal evolution of the ASE threshold for thin films of Aβ42 seeded with CSF samples from four different patients. The solutions were incubated at 37 °C before drop-casting at a given time. Black squares represent values obtained by averaging results of three consecutive experiments, and the error bars correspond to the range of values obtained in these experiments (Figure S13).

The interpretation of the ASE thresholds obtained for Aβ42 seeded with CSF was carried out in respect to the data obtained for pristine Aβ42 (Figure 3b). The ASE thresholds measured in pristine Aβ42 oligomers are in the range of 0.5–1.5 mJ/cm^2^ in the nucleation phase which lasts 90–100 min and the ASE thresholds for fibrils are between 2.7–3.5 mJ/cm^2^ in the elongation phase, which begins approximately 110 min after the start of the protein incubation (for details, see the section on the ASE-based recognition of lysozyme and Aβ42 oligomers). The ranges of ASE thresholds for oligomers and fibrils detected in pristine Aβ42 are marked in Figure 5 as yellow and red areas, respectively.

In seeded Aβ42, the ASE thresholds remained in the range corresponding to oligomers (yellow areas in Figure 5) for up to 3 days of incubation. Seeded samples incubated for at least 4 days had a distinctly higher ASE thresholds and most of them were in the range corresponding to fibrils (red areas). It is notable that, whereas the thresholds are similar for pristine and CSF-seeded Aβ42, the aggregation kinetics is significantly slower for the latter. An analogous experiment, in which incubation at an elevated temperature was replaced with agitation, was also carried out (agitation is a popular approach to accelerate the protein conversion into aggregates^53,54^). In the case of Aβ42 seeded with CSF under continuous agitation, the ASE thresholds were in the range of 3–4 mJ/cm^2^, indicating formation of fibrils, after approximately 24 h. As expected, agitation shortened the time needed to form fibrils in comparison to incubation at 37 °C; however, this time is still significantly longer than in the case of pristine Aβ42 (approximately 2 h).

Apparently, the aggregation of the recombinant protein is significantly slower in CSF-seeded Aβ42 than in pristine Aβ42. The kinetics of the ASE threshold indicate that the prefibrillary oligomers are formed immediately upon seeding with CSF, and the oligomer isoforms are stable for a surprisingly long time (days) before mature fibrils are formed. We propose two plausible explanations of this effect.

In the first place, it could be related to Aβ/Tau interactions and a formation of hybrid aggregates.^51,55^ The formation of hybrid aggregates can be extended in time due to a multistep reaction occurring between both proteins present in CSF. Therefore, the multiplication of recombinant Aβ42 oligomers can last significantly longer. We note that the increase of the ASE thresholds for ThT-stained thin films was recorded at slightly different times for seeding with CSF of different patients (the rise of ASE thresholds was observed between the third and the fourth day of the incubation at the elevated temperature (the gray area in Figure 5)). Thus, the formation of hybrid fibrils (assuming that they are formed) possibly depends on the individual characteristics of the patient’s CSF. It is also worth noting that recombinant proteins seeded with the patient’s CSF could form fibrils that do not exactly resemble the structure of the CSF aggregates.^56,57^ The reason for that are post-translational modifications (PTMs) that may be present in the CSF seeds, but not in the recombinant proteins.^58^ It was shown that some of the Aβ PTMs can significantly inhibit the formation of fibrils and can stabilize intermediate oligomers for a long period of time.^59,60^

The second hypothesis explaining the observed long aggregation time in the nucleation phase, as compared to pristine Aβ42, refers to the protective mechanisms of Tau.^61,62^ Specific Tau isoforms in CSF could act against Aβ42 aggregation and slow down the aggregation process in the nucleation phase.

Although both presented hypotheses are highly speculative, the observed variation of the ASE threshold kinetics proves that the ASE-based methodology has a great potential for identification of isoforms of protein oligomers formed during the nucleation phase in the patient’s samples.

## Conclusions

In summary, we have demonstrated that the amplified spontaneous emission (ASE) generated in samples stained with Thioflavin T (ThT) can be used for the detection of prefibrillary aggregates prepared in vitro in test tubes as well as in CSF-seeded proteins and homogenized brain tissue with oligomer phantoms. Our results prove that the ASE-based methodology has great potential for the identification of isoforms of protein oligomers formed during the nucleation phase in patient’s samples. The key parameter of ASE, the threshold for light amplification, depends both on the excited-state lifetime of ThT bound to oligomers and the yield of scattering coming from the aggregated protein. Thus, ASE is not affected by unbound ThT molecules with an ultrashort excited-state lifetime, but is sensitive to the protein structure.

Since no light amplification was obtained in ThT-stained Tau aggregates, the ASE-based technique can help to unravel the role of Tau in Aβ42 and other amyloid-β oligomerization. Our proof-of-concept experiments on the applicability of ASE in studies of neurodegeneration show the great potential of this method and suggest that it can detect amyloid oligomers in biosamples and pathogenic Aβ42 isoforms when appropriate proteins are seeded with Alzheimer’s patients’ CSF. Potentially, it can be the method of choice for distinguishing between amyloidosis (amyloid-β) and taupathy (Tau tangles). The ability of targeting selectively just one of these molecules can help in the successful neutralization of the toxicity of the other one and improve current therapeutic strategies for the treatment of the Alzheimer’s disease before its clinical symptoms occur.

The results obtained with ThT stand out when one notes that it is a histological dye used as the primary marker for characterizing amyloids in clinical research. Implementation of ASE-based techniques will not require significant changes of protocols for the analysis of biosamples. Moreover, recent technological developments such as microlasers based on whispering-gallery modes^63^ or Bose–Einstein condensates^64^ give hope that similar improvements can be applied for the detection methods based on ASE in ThT-stained amyloids. Then they could significantly improve the detection sensitivity to the extent that would allow reaching the physiological concentrations of amyloid aggregates. Without a doubt, the extension of the existing fluorescence methodology, by using laser excitation and measurements of the emitted light intensity for ThT-stained samples, could be used for a more accurate detection of oligomeric amyloids. However, we also note that the presented results demonstrate the ASE generation only with ThT-stained solid-state samples, but ASE was not observed in liquid samples using the simple experimental setup presented in this Article. It indicates that an advanced optofluidic lasing system may be required to fully elucidate ThT–protein interactions and the impact of various environments on oligomer heterogeneity.^65^

The ASE-based detection should provide access to more detailed investigation protocols of toxic species and allow a deeper insight into protein aggregation pathways at the prefibrillar stage. We envisage that our discovery will help to fully resolve the prefibrillary aggregates that are considered to be toxic and responsible for initiating numerous devastating neurodegenerative diseases.

## Supporting Information Available

Materials, preparation protocols, and methods descriptions; FTIR spectra of lysozyme, Aβ42, and insulin measured at various times of aggregation at pH values in the range of 2–12; ASE characterization of ThT thin films in poly(vinyl alcohol), and optimization of experimental parameters for protein aggregation studies; The relation between film sample thickness and ASE thresholds; ASE spectra for ThT-stained thin films of lysozyme, Aβ42, and insulin; The three series of ASE thresholds experiments; Results of coherent backscattering experiments with films made of lysozyme aggregates (PDF)
