# Supplementary material for: Laser Emission of Thioflavin T Uncovers Protein Aggregation in Amyloid Nucleation Phase
Source: ACS Photonics. 2021 Aug 11;8(9):2598–609. doi: 10.1021/acsphotonics.1c00082 (PMC8451393; doi:10.1021/acsphotonics.1c00082)
Supplement: Supplementary file 1 — ph1c00082_si_001.pdf [file ph1c00082_si_001.pdf]

Supporting Information

Laser Emission of Thioflavin T Uncovers Protein Aggregation in Amyloid Nucleation Phase

Piotr Hanczyc* & Piotr Fita

Institute of Experimental Physics, Faculty of Physics, University of Warsaw, Pasteura 5, 02-093 Warsaw, Poland

Number of pages: 19

Number of Figures: 13

Number of tables: 4

Materials

Thioflavin T: Ultrapure grade Thioflavin T (ThT) was purchased from AATBioquest. A set of ThT solutions with concentrations of 0.11, 0.21, 0.32, 0.44, and 0.85 mM was prepared for the characterization of the dye in polyvinyl alcohol (PVA) films and for the determination of the optimal ThT concentration for ASE generation in presence of proteins. Concentrations of ThT solutions were assessed on the basis of absorbance at 412 nm with the molar extinction coefficient assumed as 36,000 $M^{-1}cm^{-1}$.[1]

Polyvinyl alcohol: Polyvinyl alcohol (PVA) of molecular weight 89,000-98,000 was purchased from Sigma-Aldrich and used as obtained. For film samples preparation the PVA powder was dissolved in distilled water at 10% concentration (w/v) by stirring the mixture for 5 min at room temperature. The PVA solution was then incubated at 80ºC for 30 min under continuous stirring until a transparent viscous solution was obtained. Samples containing PVA and ThT were prepared by mixing the two constituents at a volume ratio of 10:1, using aqueous dye solutions of concentrations 0.11, 0.21, 0.32, 0.44, and 0.85 mM. Typically 250 µl of the PVA-ThT solution was drop casted. The thin films were dried in ambient conditions.

Insulin: Insulin monomer protein from bovine pancreas was purchased from Sigma-Aldrich. The protein was dissolved to 1.0 mM concentration at pH 2 in 0.003 mM H_2SO_4 and at pH 12 in $Na_2B_4O_7$-KCl-NaOH buffer. The solutions were filtered through a 0.45 µm filter and the sample at pH 2 was incubated at 65°C for 24 h (the sample at pH 12 was not heated and a thin film was prepared in ambient conditions). To that insulin solutions 0.2 mM ThT solution was added at a volume ratio of 1:20 (insulin:ThT solutions). 200 µl of the insulin-ThT mixture were drop casted on glass slides and allowed to dry for 10 minutes at a hot plate with temperature adjusted to 65ºC.

Lysozyme: lysozyme protein from chicken egg white was purchased from Sigma-Aldrich. The protein was dissolved at a set of concentrations: 0.07, 0.16, 0.32, 0.70, 1.10, and 1.5 mM at pH 2 in 0.003 mM H_2SO_4 and filtered through a 0.45 µm filter. The solutions were incubated at 65°C. After 8 days viscous liquids were obtained indicating formation of lysozyme fibrils. The aggregation process was monitored by the ThT fluorescence assay in the solutions. Thin films were prepared by drop casting 250 µl of the solutions containing ThT at concentrations 0.11, 0.21, 0.32, 0.44, and 0.85 mM and lysozyme aggregates at concentrations 0.07, 0.16, 0.32, 0.70, 1.10, and 1.5 mM. In each case the volume ratio was kept at 1:20 (ThT : Lysozyme). For more details see the section: *ASE characterization of ThT thin films and optimization of parameters for protein aggregation studies*.

Aβ42: Human *A*β42 peptide was purchased from GenScript and used as obtained (purity >95%). The protein was dissolved in water at a concentration of 1.38 mM. The solution was incubated at 37°C for 10 h. In a procedure similar to that described above for the other proteins, aggregation was monitored by the ThT fluorescence assay (the results are shown in Fig. S5). For the thin film samples preparation, 48 µl were collected from the Aβ42 stock solution at the specific time of its aggregation. To that Aβ42 solution 3µl of 0.2 mM ThT were added (volume ratio 1:16). The solutions were drop casted on glass slides and dried for 20 minutes at a hot plate at 37ºC.

Tau: Tau protein was purchased from rPeptide and used as obtained (purified using SDS-PAGE >90%) . The protein was dissolved in water at a concentration of 1.36 mM. 200 µg/ml of heparin (obtained from Sigma-Aldrich) was added to the solution in order to initiate Tau aggregates formation. The solution

was incubated at 37°C for 2 weeks and the ThT fluorescence assay was applied periodically. The increase of the ThT fluorescence was observed after 5 days of incubation. Thin films containing Tau and ThT were prepared using analogical methodology as for the films made of Aβ42-ThT. In brief, 48 µl of Tau solution were collected from the protein stock solution at specific times of its aggregation. To that Tau solution 3µl of 0.2 mM ThT were added (volume ratio 1:16). The solutions were drop casted on glass slides and dried for 20 minutes at a hot plate at 37°C.

CSF samples: CSF samples were de-identified, archived leftover aliquots of CSF collected from patients with the Alzheimer disease, following a procedure approved by the Ethical Committee at the University of Gothenburg (reference number: EPN 140811). CSF samples were seeded with Tau and Aβ42 at a volume ratio of 1:12 whereby typically 4 µl of undiluted CSF were mixed with 48 µl of 1.36 mM Tau or 1.38 mM Aβ42 solutions. Protein-CSF mixtures were incubated at 37°C for 2 weeks in the case of Tau and for 5 days in the case of Aβ42. At a specific time of incubation the protein-CSF solution was collected. To that solutions 3 µl of 0.2 mM ThT were added so the volume of the entire sample was equal to 55 µl. ThT-stained protein-CSF samples were drop casted on glass slides and allowed to dry for 20 minutes at 37°C.

Brain tissue: Fresh bovine brain tissue was purchased at a grocery store and doped with amyloid phantoms of Aβ42. The Aβ42 phantoms were prepared according to the protocol presented above for the Aβ42 aggregation. 48 µl of the protein aggregates solution prepared in a test tube were collected. The collected Aβ42 solution was mixed with the brain tissue and to that mixture 3µl of 0.2 mM ThT was injected. All constituents were vortexed for 5 minutes in order to obtain a homogenous dispersion. The homogenate was deposited on a glass slide, uniformly distributed using a pipette tip, and dried at 37°C for 20 minutes.

Methods

Fluorescence spectroscopy: Steady-state fluorescence spectra and fluorescence decay curves of ThT mixed with proteins and amyloids deposited on glass slides were recorded in custom-built experimental setups.

For recording fluorescence spectra samples were excited with light of a xenon arc lamp passing through a monochromator with the central wavelength and transmission bandwidth set to 405 nm and 16 nm, respectively, and a short-pass absorption filter (absorption edge at 450 nm). The emitted light was analysed with a SpectraPro 150 Czerny-Turner imaging monochromator equipped with a CCD camera (Andor DU420A-BU2). The fluorescence light was collected at the right angle to the direction of the excitation light and the studied thin film was oriented at 45° angle with respect to the excitation and detection directions. A long-pass filter (edge at 430 nm) in front of the monochromator was used to eliminate scattered excitation light. The recorded spectra were corrected for the transmission curve of this filter. The overall spectral responsivity curve of the apparatus was determined using secondary emission standards according to the procedure proposed by Gardecki and Maroncelli [2].

Fluorescence decays were recorded using a custom-built setup based on the PicoQuant HydraHarp 400 Multichannel Picosecond Event Timer and the picosecond diode laser PicoQuant LDH-P-C-405B working at 405 nm. Repetition frequency of the laser was set to 10 MHz. Thin film samples were oriented at approx. 45° with respect to the excitation beam, with the fluorescence light collected at the right angle to

the direction of excitation. The collected light passed through a long-pass cut-off filter in order to remove scattered excitation light. The setup was also used for measurements of liquid samples, therefore the fluorescence light passed through a polarizer, whose transmission axis was oriented at the magic angle (54.7°) with respect to the polarization of the excitation beam, in order to eliminate artifacts due to rotational diffusion. The fluorescence light was focused on the entrance slit of a Czerny-Turner monochromator (Acton Research SpectraPro 150) used to select a given wavelength from the fluorescence spectrum. After the monochromator the light was detected with the Hamamatsu R3809U-50 photomultiplier sensitive in the 160-850 nm range. The output signal from the photomultiplier was amplified with the Ortec Model 9327 amplifier prior to routing it to the HydraHarp module. The overall instrumental response function, measured by scattering the excitation light in a suspension of titanium dioxide, was narrower than 100 ps FWHM. The data were analyzed using the DecayFit 1.4 free software[3].

Attenuated Total Reflectance Fourier-transform infrared spectroscopy (ATR-FTIR): IR spectra in solid thin films were collected using Nicolet iS50 FT-IR spectrophotometer equipped in smart iTR ATR accessory with a diamond crystal (Thermo Scientific). The penetration depth of the IR beam ranged from 1 to 10 µm. The film samples were put on the top of the diamond crystal. After collecting the IR spectra for a film sample the ATR correction was performed: the spectrum collected for a pure glass slide was subtracted from the spectrum of the film sample deposited on the same glass type in order to obtain the film IR spectrum. The spectral resolution was 4 cm^{-1} and, typically, 32 scans were averaged for a single spectrum.

Transmission electron microscopy (TEM): Lysozyme samples were plunge-frozen onto Quantifoil R2/2 holey carbon grids using a Thermo Fisher Vitrobot. 2D electron cryo-images were taken on a Thermo Fisher Glacios TEM operating at 200 kV capable of resolving structures down to 3 Å. The microscopy system was equipped with a 4k x 4k Falcon 3EC direct electron detection camera for visualizing biosamples.

Amplified spontaneous emission (ASE): Spectra of amplified spontaneous emission of ThT in thin films were recorded in the experimental setup shown schematically in Scheme 1.

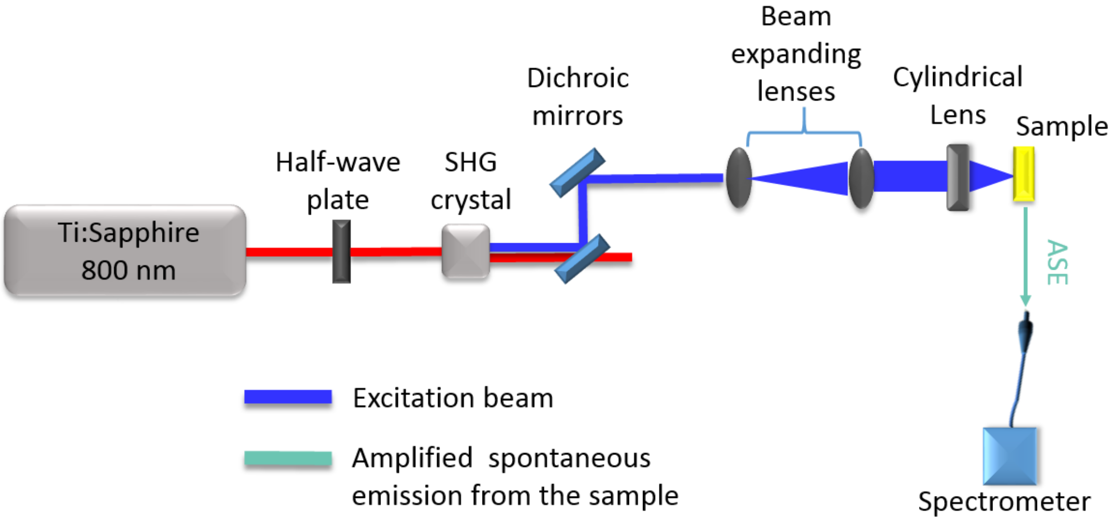

Scheme 1 Experimental setup for the analysis of the amplified spontaneous emission (ASE) of ThT in thin films.

Studied films were excited at 400 nm with femtosecond pulses obtained by frequency doubling the output beam of a Ti:Sapphire femtosecond amplifier system (Legend Elite Duo) working at 5 kHz repetition rate. A beta-barium borate (BBO) crystal 1 mm thick was used to generate the second harmonic (SH) light of the amplifier output beam. Energy of the resulting SH pulses was adjusted by rotating the half-wave plate mounted in front of the BBO crystal. The SH light was separated from the remaining fundamental light by a pair of dichroic mirrors. The maximum energy of an SH pulse achievable in this configuration was approximately 300 μJ. After the dichroic mirrors the beam was first expanded with a telescope formed by a pair of spherical lenses with focal lengths of -50 and 125 mm. Then it was focused with a cylindrical lens in order to form a narrow stripe of light at the position of the film sample. The profile of the beam in the focal point was determined with the Thorlabs LC1-USB line CCD camera. It was approximately Gaussian in both directions with the full width at half maximum (FWHM) of (7.7±0.3) mm in the horizontal and (17±1) μm in the vertical direction, respectively. The area of the focal point at the FWHM intensity level, used for the calculation of average intensities of the excitation light reported in the current work, was taken as (0.0013±0.0001) cm^2. In some cases the maximum excitation intensity obtainable in the above described configuration was insufficient to generate ASE. In such a case the cylindrical lens was replaced with a spherical one in order to reduce the size of the focal point and only qualitative experiments were carried out (absolute values of the excitation intensity were not measured in the configuration with the spherical lens).

The studied films were oriented perpendicularly to the direction of the excitation beam. The amplified spontaneous emission (ASE) was detected in the plane of the films, along the direction of the elongated linear focus of the cylindrical lens. The ASE light was collected with a fiber and delivered to the Ocean Optics USB 2000 spectrometer, whose spectral resolution was around 4 nm. For the determination of ASE thresholds the intensity of the excitation light was gradually increased by rotating the half-wave plate and the spectrum of the emitted light was simultaneously monitored. Plots of the dependence of the light intensity at the wavelength corresponding to the maximum of the ASE spectrum on the excitation intensity were used to determine the ASE generation thresholds.

1. Groenning, M., Binding mode of Thioflavin T and other molecular probes in the context of amyloid fibrils—current status. *Journal of chemical biology* **2010,** *3* (1), 1-18.
2. Gardecki, J.; Maroncelli, M., Set of secondary emission standards for calibration of the spectral responsivity in emission spectroscopy. *Applied Spectroscopy* **1998,** *52* (9), 1179-1189.
3. DecayFit - Fluorescence Decay Analysis Software 1.4, FluorTools, www.fluortools.com.

Lysozyme aggregates characterization in solution and solid films

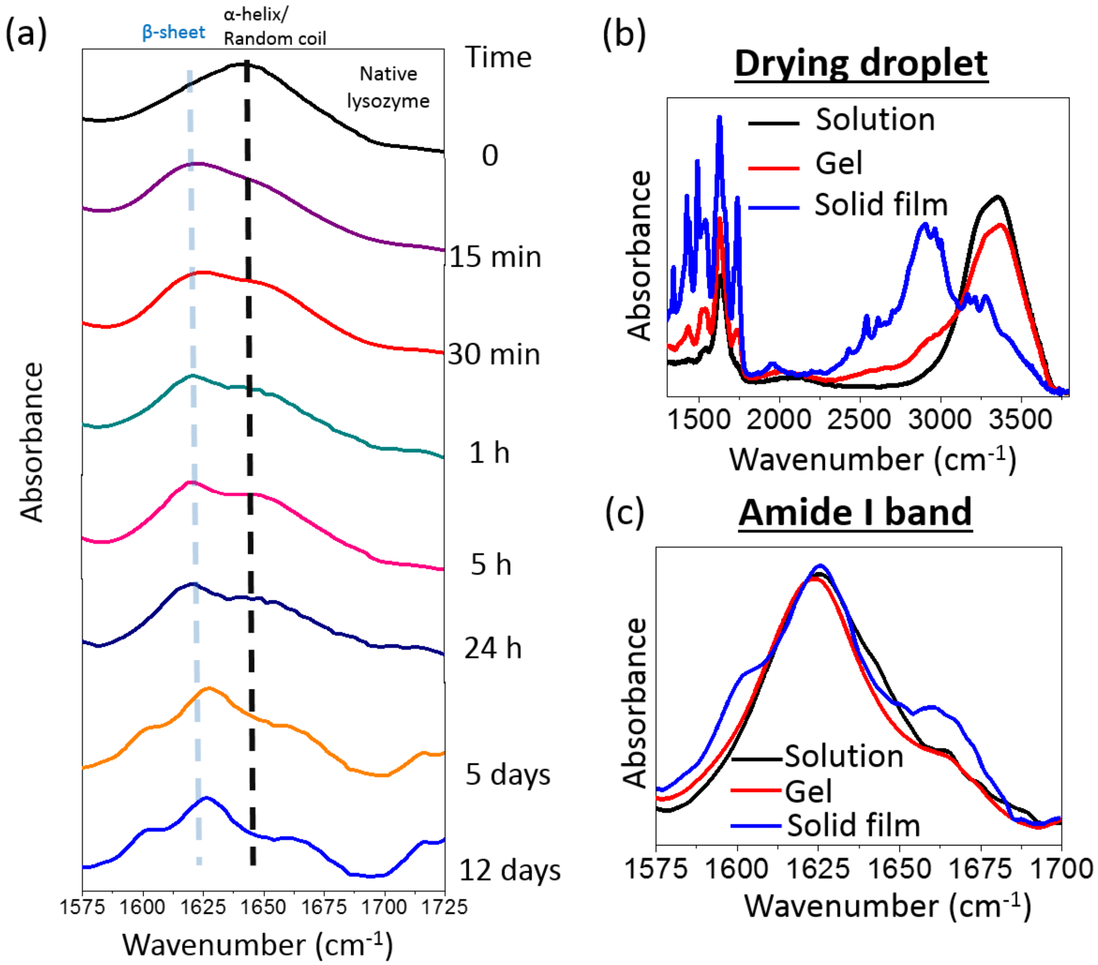

Fig. S1 (a) ATR-FTIR spectra of solid films of lysozyme protein deposited on glass slides at the specific time of solution incubation at 65ºC. (b) ATR-FTIR spectra of a drying droplet containing lysozyme fibrils after 5 days of incubation at 65ºC. The spectra were collected in a solution, gel and solid film. (c) The amide I band of a drying droplet with the maximum at 1622 cm^{-1} corresponding to the β-sheet structure that was recorded in a solution, gel and solid film.

ASE characterization of ThT in thin films and optimization of parameters for protein aggregation studies

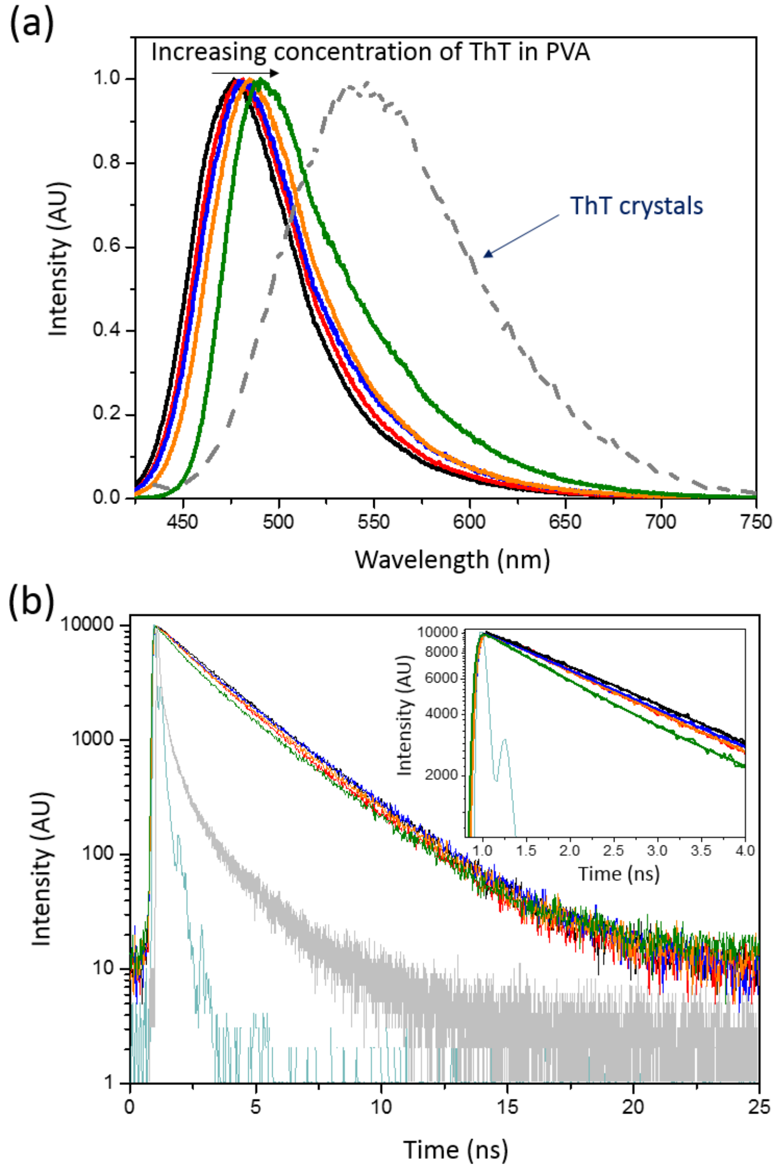

Fig. S2 (a) Steady-state spectra and (b) fluorescence decays of ThT in PVA films at five dye concentrations (concentration values for solutions before drop-casting the ThT/PVA mixtures are given), 0.11 mM (black), 0.21 mM (red), 0.32 mM (blue) 0.44 mM (orange) and 0.85 mM (green) and of ThT crystals (grey) drop casted on glass slides. Instrument response function is shown in cyan. The inset presents the decays in time up to 4 ns with the monoexponential fitting curves.

At first, optical properties of ThT in condensed phase were characterized in poly(vinyl alcohol) (PVA) in order to determine the optimal ThT concentration for investigation of protein aggregation by ASE. The experiments were conducted in thin PVA films and ThT concentrations examined were equal to 0.11, 0.21, 0.32, 0.44, and 0.85 mM (concentrations of the dye solutions before mixing them with PVA and drop casting thin films). In Fig. S2(a) fluorescence spectra recorded for ThT embedded in PVA thin films are shown. The spectra became gradually red-shifted with the increasing dye concentration. This effect indicates that the polymer becomes gradually saturated with the increasing ThT concentration and the dye excess crystallizes.

The same samples were studied by time-resolved fluorescence spectroscopy (Fig. S2(b)). Fluorescence decays were collected at 490 nm and were monoexponential with lifetimes between 2.17 ns and 2.36 ns for ThT concentrations up to 0.44 mM, with no systematic dependence of the lifetime on the dye concentration (Table S1). This fact indicates that dye molecules were homogenously dispersed in the polymer matrix and no significant effects of the dye aggregation appeared up to this concentration level. Only at the highest studied ThT concentration, 0.85 mM, a slightly shorter fluorescence lifetime of 2.03 ns was measured, which most probably reflects the presence of a noticeable amount of ThT crystals in PVA at this concentration.

Table S1 ThT fluorescence lifetimes in PVA

ThT concentration (mM)	Lifetime (ns)
0.11	2.36±0.23
0.21	2.17±0.20
0.32	2.34±0.32
0.44	2.24±0.21
0.85	2.03±0.15

Next, PVA thin films prepared at the same set of ThT concentrations were examined by ASE. Fig. S3(a) shows spectra of the emitted light, which at a low excitation intensity corresponded to ThT fluorescence. With the increasing excitation intensity a gradual spectral narrowing was observed, which reflected the transition from fluorescence to ASE. The excitation intensity value at the transition point between fluorescence and ASE is the ASE threshold (Fig. S3(a)). The ASE threshold was approximately 1 mJ/cm^2 at concentrations of 0.11 mM and 0.21 mM (for lower dye contents in PVA no ASE was observed). The further increase of the ThT concentration up to approx. 1 mM resulted in a sharp decrease of ASE thresholds. At even higher dye contents (>1.2 mM) ThT crystallized in PVA and no ASE was observed in such samples.

On the basis of steady-state and time-resolved fluorescence studies and ASE experiments carried out in PVA the ThT concentration optimal for examination of protein aggregation was determined to be approx. 0.2 mM. In order to independently confirm this finding, in the next step PVA was replaced by lysozyme aggregates and thin films were prepared of lysosome/ThT mixtures. Steady-state and time-resolved fluorescence measurements were subsequently conducted (Fig. S4). The ThT fluorescence decays

were registered at the same ThT concentration range as used before (from 0.11 mM to 0.85 mM). The lysozyme concentration was kept constant at 1.5 mM. The fluorescence decays had a biexponential character (Fig, S4(b)) and the increase of the dye concentration resulted in a significant shortening of the fluorescence lifetime (Table 2). This observation indicates that ThT at mM concentration range saturates the binding sites of the protein and increase of the dye concentration leads to a significant crystallization of the excess dye molecules. The biexponential character of the decays suggests that there are two ThT species in thin film samples made of protein aggregates. Most probably they can be identified with molecules bound to the protein and crystallized molecules.

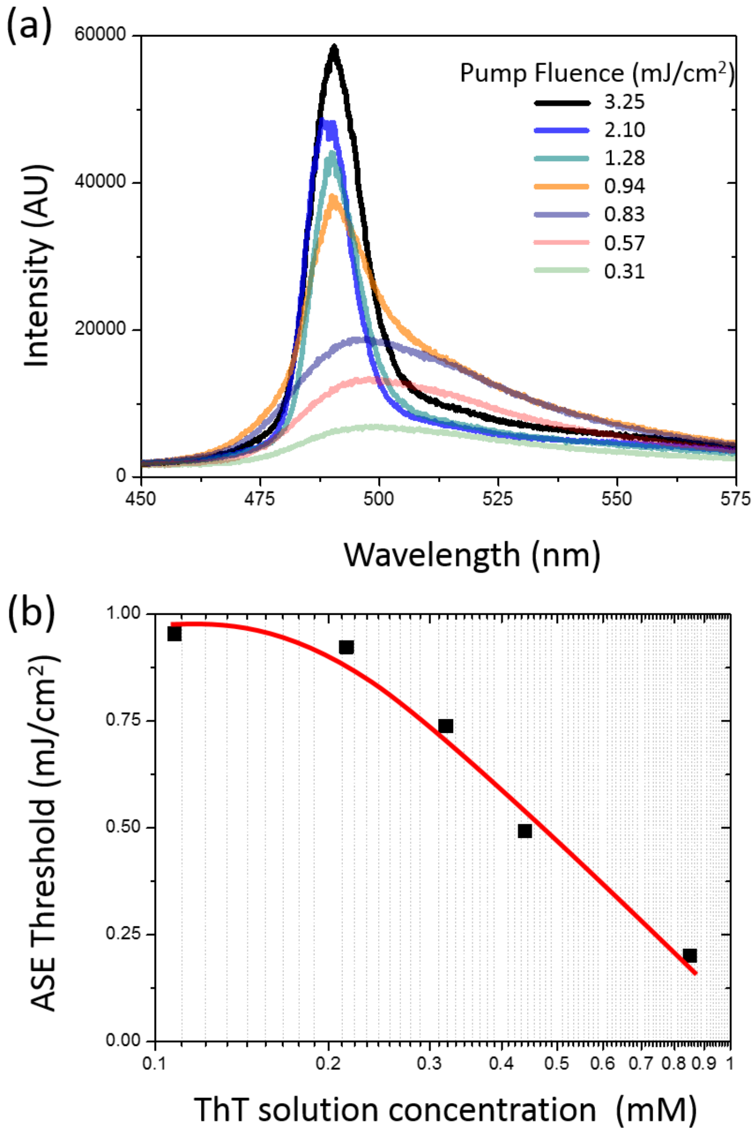

Fig. S3 a) The dependence of emission spectra of ThT in PVA on the excitation intensity, which shows spontaneous emission (fluorescence) below approx. 0.9 mJ/cm^2 and ASE above this threshold. b) The dependence of ASE thresholds on ThT concentration in PVA (ThT solution concentrations were 0.11, 0.21, 0.32, 0.44, and 0.85 mM).

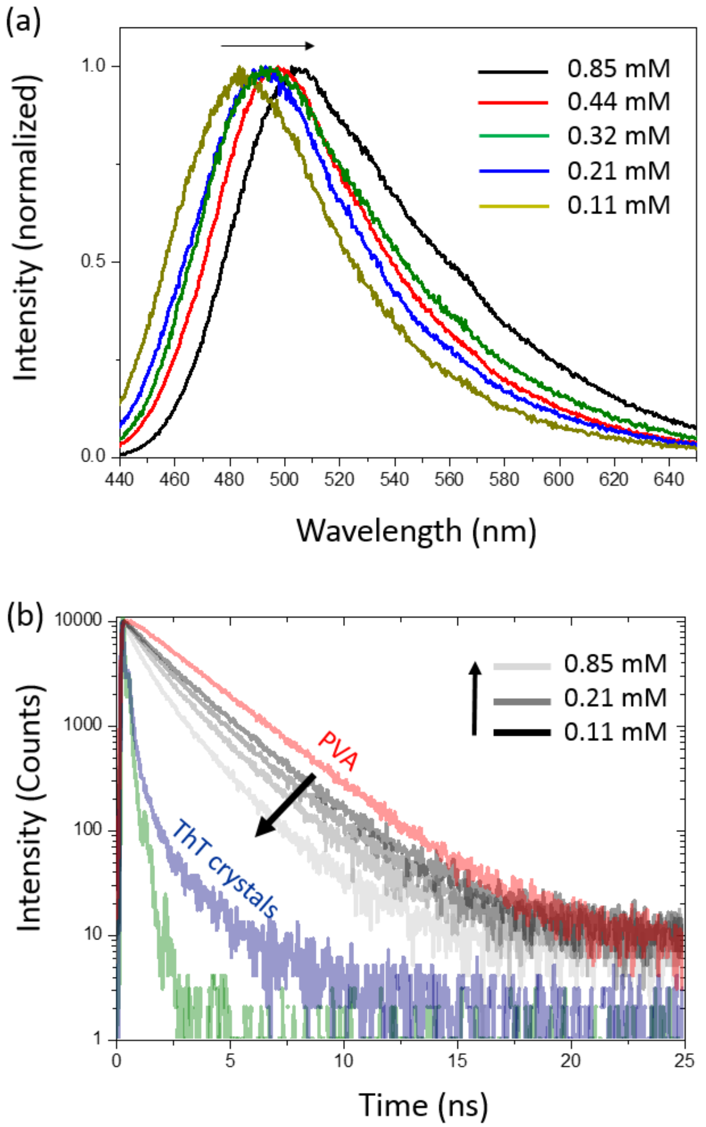

Fig. S4 (a) Steady-state fluorescence spectra of ThT-stained lysozyme protein aggregates recorded for a set of films with the increasing dye concentration and (b) Fluorescence decays recorded for ThT embedded in PVA (red), in lysozyme protein aggregates (colors from black to light gray correspond to the increasing dye concentration resulting in the shortening of the fluorescence lifetime), and for ThT crystals drop casted on a glass slide (blue). All samples were in the form of solid thin films. The lysozyme protein solution had a concentration of 1.5 mM prior to drop casting. The instrumental response function is plotted in light green.

Table S2 ThT fluorescence lifetimes in presence of lysozyme aggregates (biexponential fluorescence decays were recorded)

ThT concentration (mM)	Lifetimes (ns)
0.11	1.67±0.12 & 2.89±0.32
0.21	0.90±0.05 & 2.13±0.17
0.85	0.69±0.03 & 1.82±0.13

Finally, the optimal protein concentration was found. For this purpose a series of experiments, in which the ThT concentration was kept constant at 0.21 mM and the lysozyme aggregates concentration was gradually decreased starting from 1.5 mM was carried out. The lower the protein concentration is, the less binding sites are available for ThT molecules and the thinner films are formed. The film thickness was found to be linearly proportional to the protein concentration (Table 3, Fig. S5). The ASE threshold, in turn, significantly drops down when the lysozyme concentration increases from 0.035 to 0.70 mM. At lower protein concentrations a significantly higher excitation intensity is necessary to obtain ASE.

In conclusion, it was found that the optimal ThT concentration for studying protein aggregation by ASE is in the range of 0.1 mM - 0.25 mM. In this concentration range all available binding sites in protein aggregates are occupied and ASE is efficiently generated. On the other hand, the optimal protein concentration was determined to be between 0.75 mM and 1.5 mM, which corresponds to the film thickness in the range of 30-50 μm.

Table S3 Dependence of the thin film thickness on lysozyme concentration

Lysozyme Concentration (mM)	Thickness (μm)
1.50	50
1.10	40
0.70	30
0.32	22
0.16	18
0.07	15
0.035	12

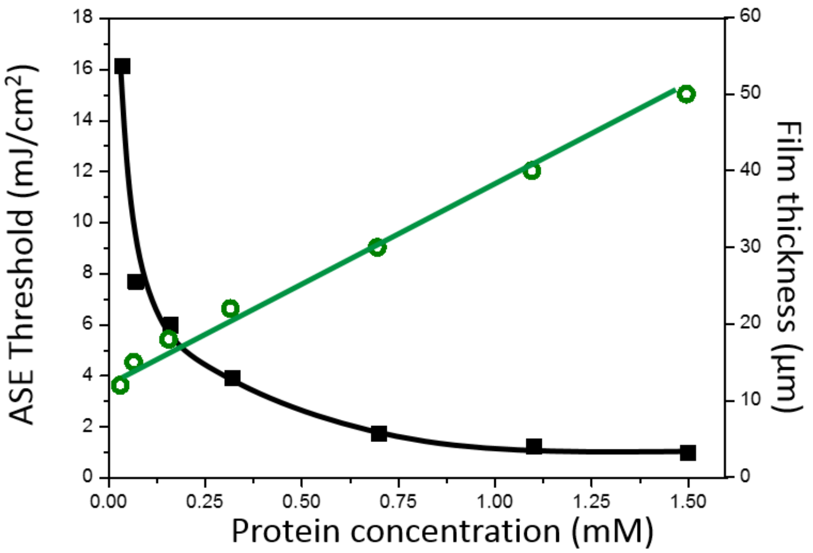

Fig S5 Dependence and the trend line of the ASE thresholds (black) and film sample thickness (green) on the lysozyme concentration. ThT concentration was equal to 0.21 mM.

Additional experimental data

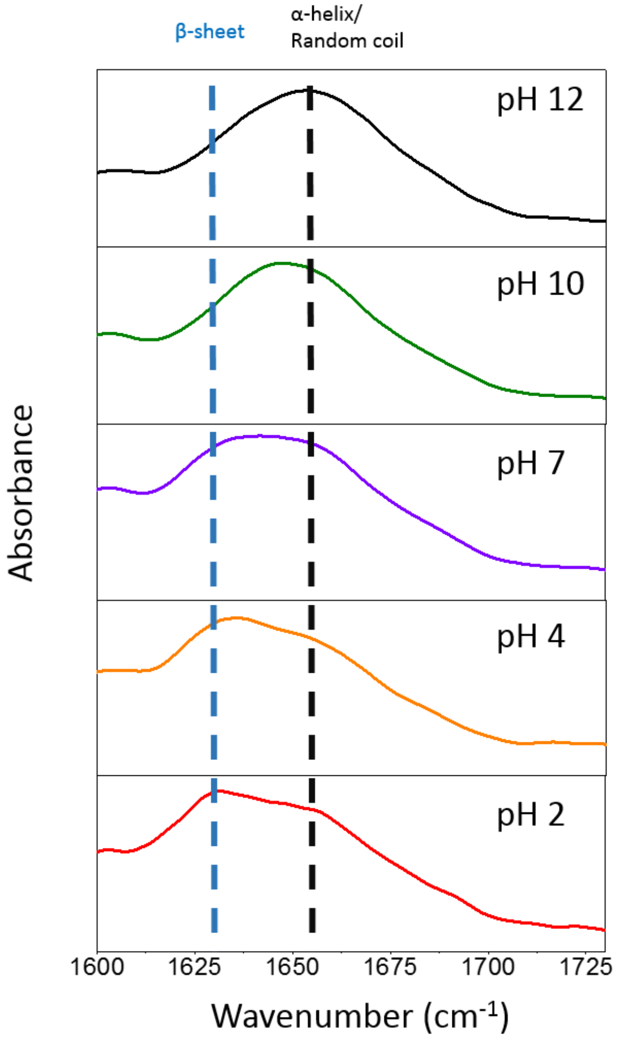

Fig. S6 ATR-FTIR spectra of thin films of the insulin protein deposited on glass slides from solutions prepared at the pH range of 2 – 12 and incubated for 15 min at 65ºC.

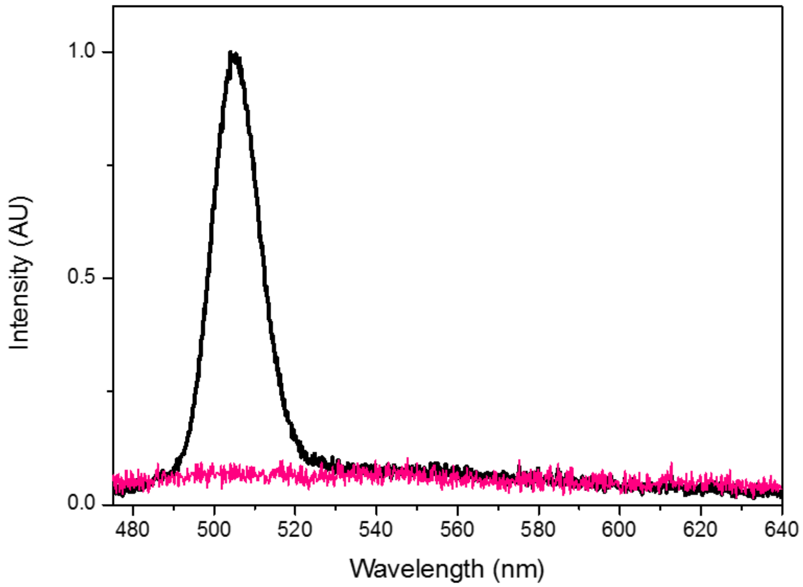

Fig. S7 Emission spectra recorded in ThT-stained thin films drop casted from a solution containing insulin fibrils formed at pH 2 (black) and monomeric insulin dissolved at pH 12 (pink). ASE is generated in presence of fibrils, whereas only weak fluorescence can be detected in thin films made of the monomeric protein.

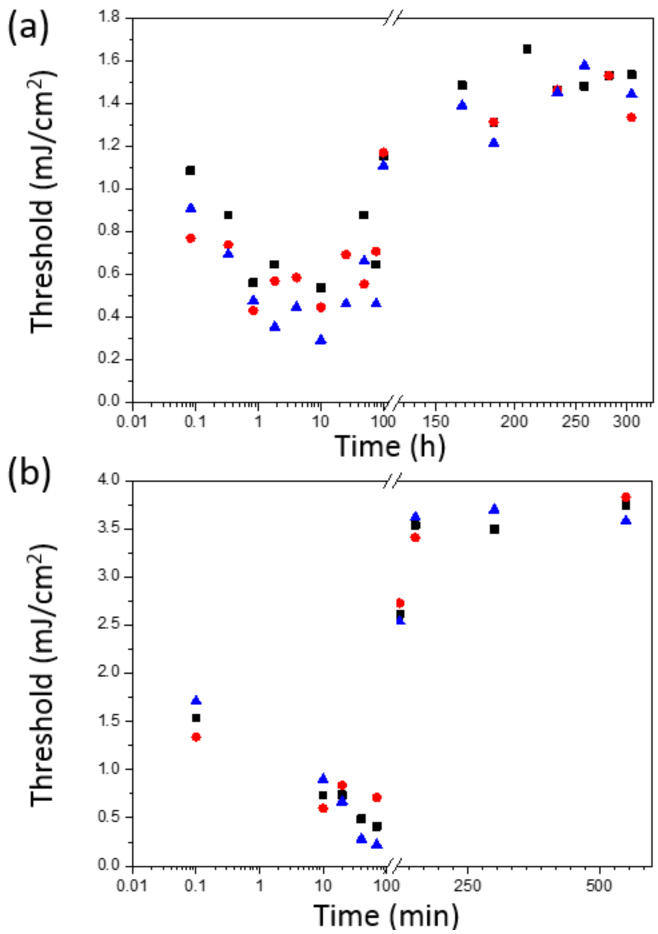

Fig. S8 Three series of ASE thresholds measured in solid thin films at different times of protein incubation at 65ºC in the case of lysozyme (a) and at 37ºC in the case of Aβ42 (b). The samples were drop casted at a given time of incubation from solutions containing ThT and (a) lysozyme, (b) Aβ42.

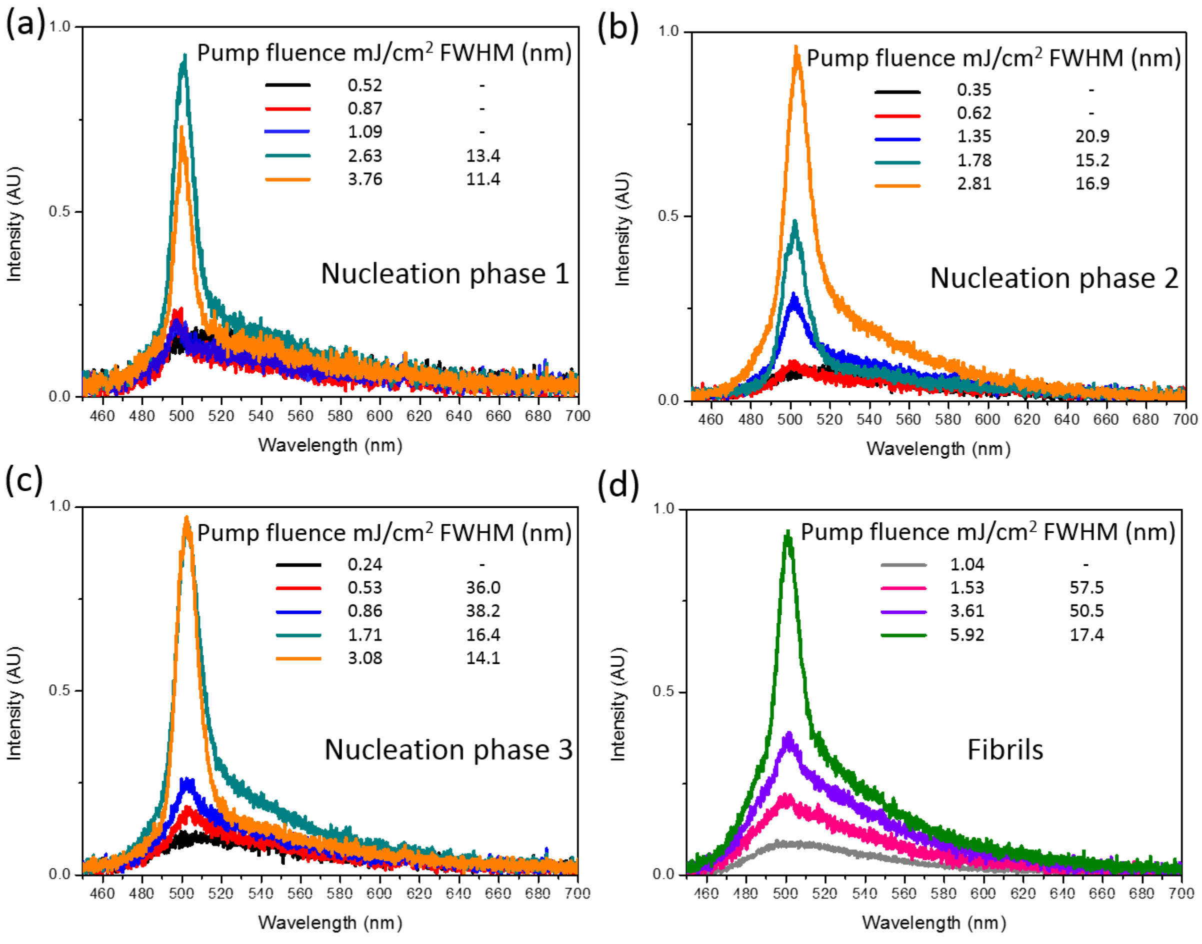

Fig. S9 The dependence of emission spectra on the excitation intensity recorded for films made of ThT-stained lysozyme aggregates. The film samples were prepared from the lysozyme protein solution incubated for times of (a) 15 min, (b) 10h, (c) 50h corresponding to the nucleation phase, and (d) 12 days corresponding to the elongation phase and the fibrils formation.

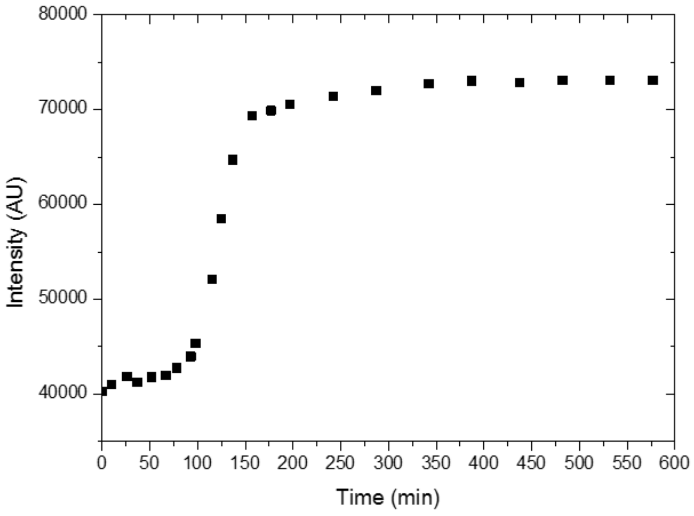

Fig. S10 Aggregation kinetics of Aβ42 obtained by monitoring ThT fluorescence intensity during incubation of the solution at 37ºC.

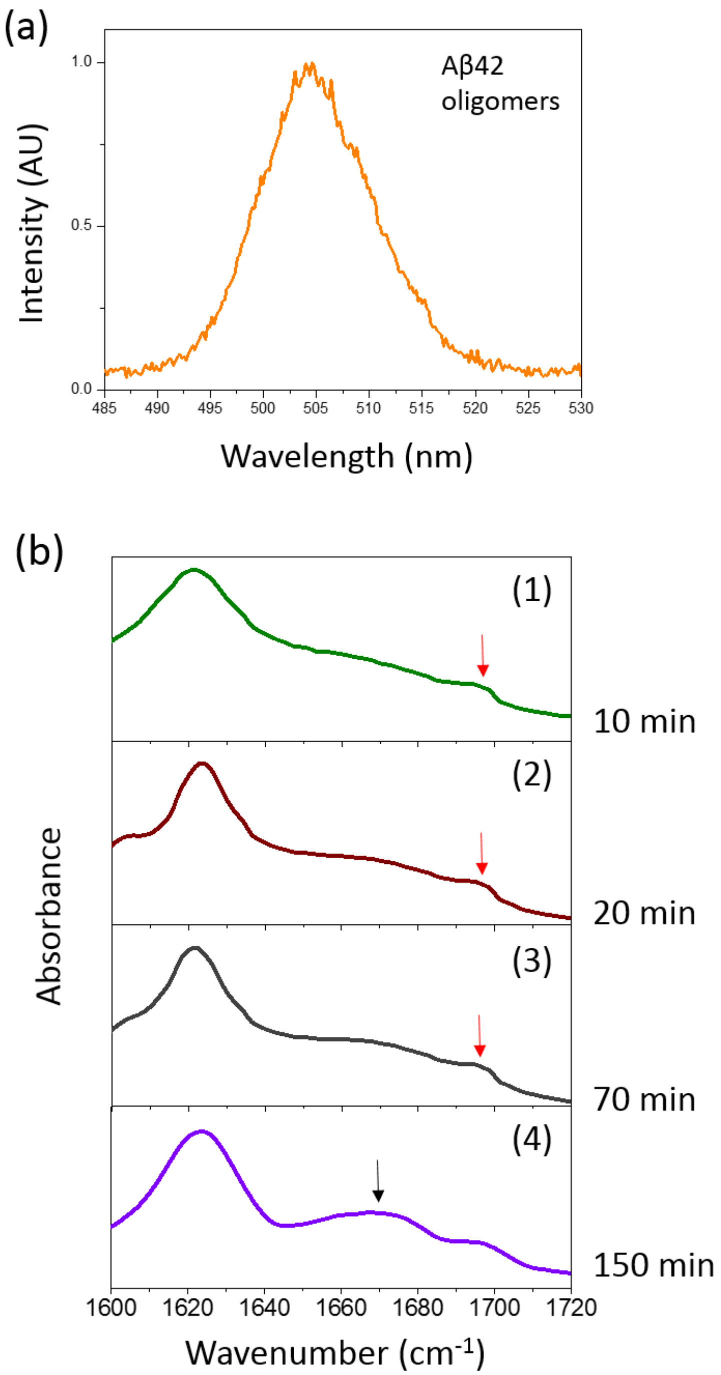

Fig. S11 The ASE spectrum for ThT-stained thin films made of Aβ42 oligomers, (b) ATR-FTIR spectra of thin films made of Aβ42 deposited on glass slides at specific times of solution incubation at 37ºC. The characteristic peak at 1695 cm^{-1} (marked with the red arrow) seen in the spectra of samples (1), (2), (3) indicates the presence of Aβ42 oligomers in the time span from 10 to 70 min. The peak at 1670 cm^{-1} marked with the black arrow indicates the formation of elongated fibrils after 150 min from the beginning of the incubation at 37ºC.

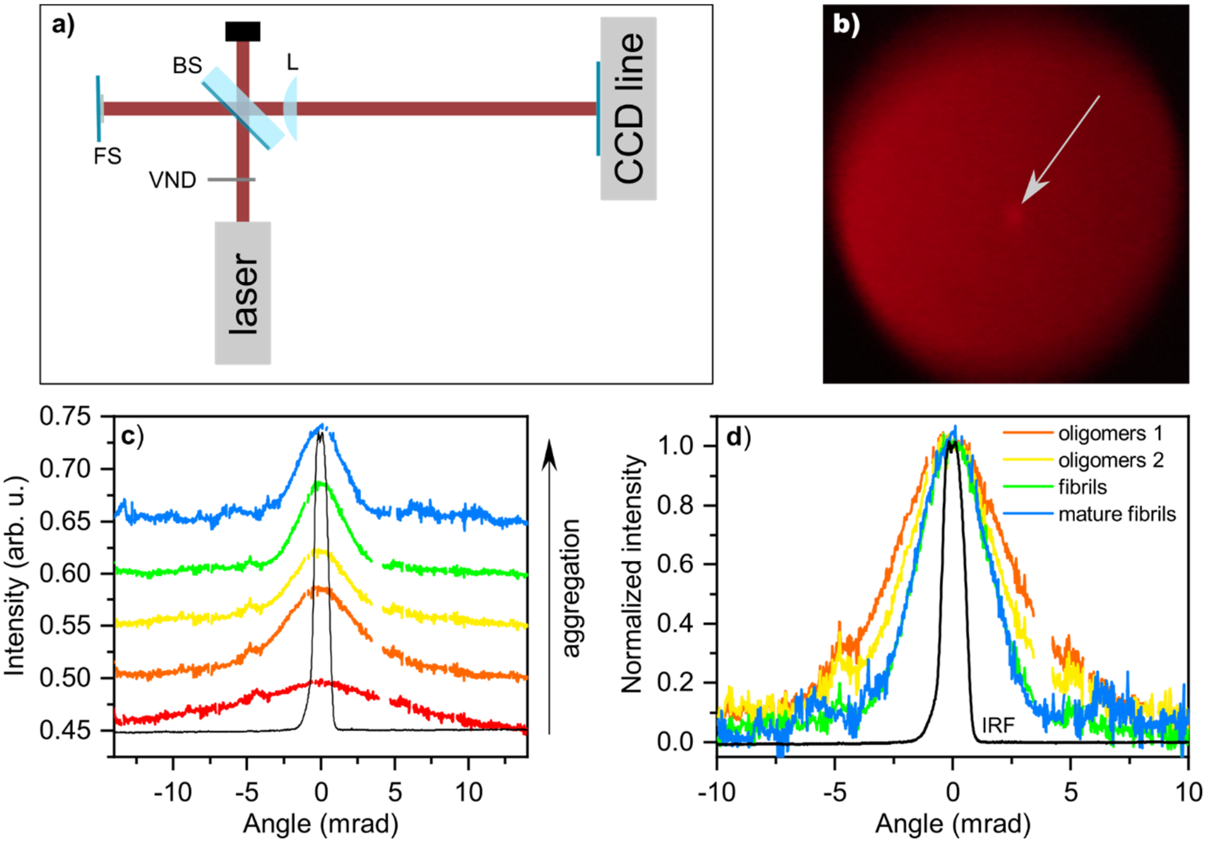

Fig. S12 Coherent back-scattering measurements of lysozyme aggregates. **a)** Scheme of the experimental setup (FS – film sample in a rotating mount, BS – beam splitter, VND – variable optical density neutral filter, L – 500 mm lens, laser - 657 nm laser diode, CCD line – Thorlabs LC1-USB HighRes line camera). **b)** Image of the coherent back-scattering spot seen on the incoherent scattering background for a film of lysozyme fibrils recorded with a photo camera. **c)** Coherent back-scattering curves for lysozyme aggregates. The degree of aggregation raises from bottom to top and each consecutive curve is shifted vertically by 0.05 for clarity: red - monomers (native protein), orange – early oligomers (nucleation phase), yellow - late oligomers (nucleation phase), green - fibrils (elongation phase), blue - mature fibrils, black – spatial instrumental response function obtained by replacing the sample with a laser mirror (not to scale with the other curves). **d)** Coherent back-scattering curves for lysozyme aggregates after normalization in order to compare their widths. Color-coding is the same as in panel c.

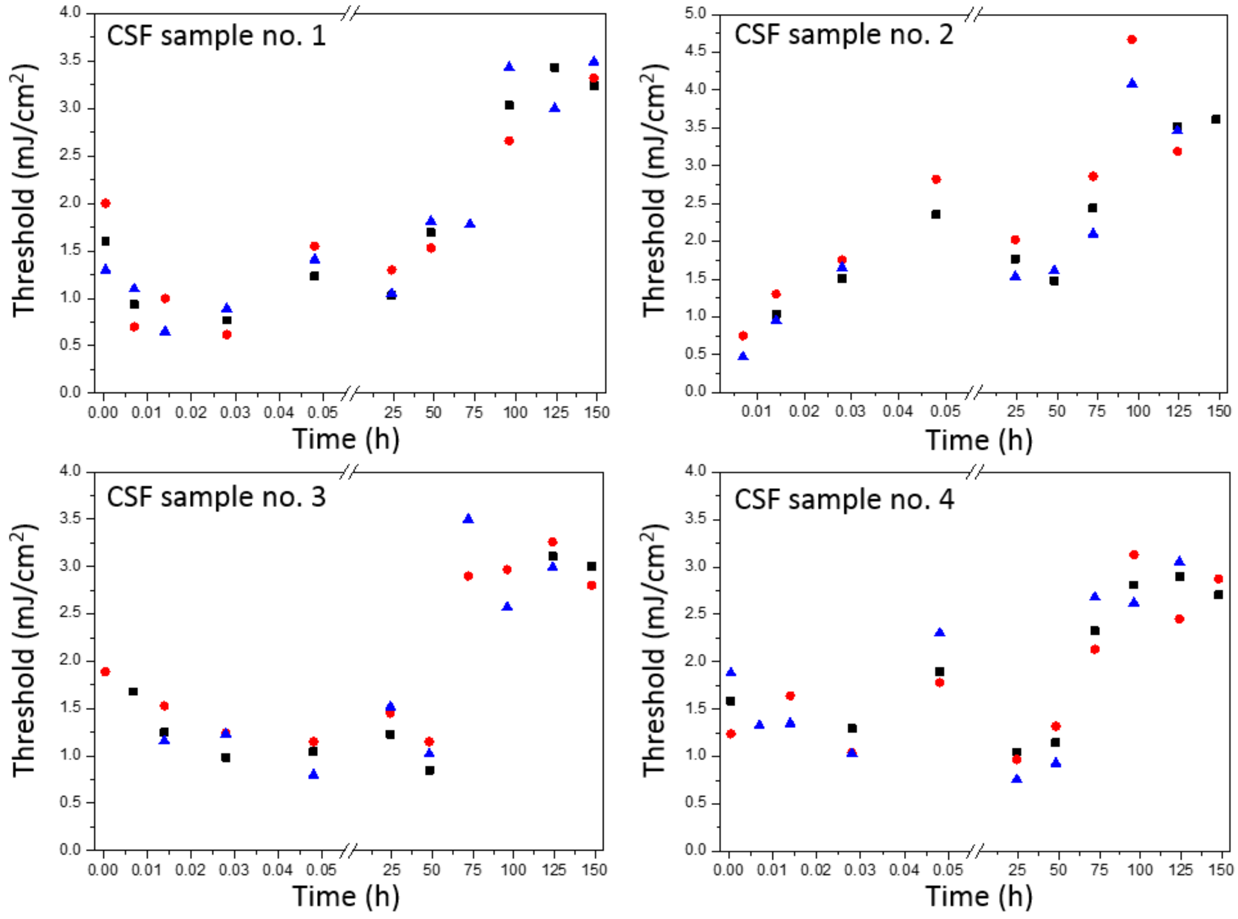

Fig. S13 Three series of ASE thresholds measured in solid thin films at different times of sample incubation. The samples were drop casted at a given time from solutions containing ThT and Aβ42 seeded with CSF collected from four patients with diagnosed Alzheimer disease.

Table S4 Concentrations of Tau, β-amyloid and phosphorylated Tau (Ptau) in four patient's CSF samples determined upon collection of the material at the University of Gothenburg (Sweden)

Sample number	Tau (ng/l)	βAm (ng/l)	Ptau (ng/l)
1	294	393	35
2	584	436	67
3	771	552	71
4	631	604	69
